# Insights into the physiological and metabolic features of *Thalassobacterium*, a novel genus of *Verrucomicrobiota* with the potential to drive the carbon cycle

**DOI:** 10.1128/mbio.00305-25

**Published:** 2025-03-20

**Authors:** Xin-Yun Tan, Xin-Jiang Liu, De-Chen Lu, Yu-Qi Ye, Xin-Yu Liu, Fan Yu, Hui Yang, Fan Li, Zong-Jun Du, Meng-Qi Ye

**Affiliations:** 1 Marine College, Shandong University 601999 , Weihai, Shandong, China; 2 Shenzhen Research Institute of Shandong University, Shenzhen, Guangdong, China; 3 Weihai Research Institute of Industrial Technology of Shandong University 727768 , Weihai, China; University of Washington, Seattle, Washington, USA

**Keywords:** PUL (polysaccharide utilization locus), dissolved organic matter (DOM), *Verrucomicrobiota*, carbon cycle

## Abstract

*Verrucomicrobiota* are widely distributed across various habitats but are difficult to culture. Some previous multiomics analyses reported that *Verrucomicrobiota* have outstanding metabolic capacity for organic matter degradation and are able to degrade and synthesize polysaccharides, two activities that could contribute significantly to the Earth’s carbon cycle. Here, we isolated from marine sediment two novel strains, *Thalassobacterium maritimum* SDUM461003^T^ and *Thalassobacterium sedimentorum* SDUM461004^T^, that represent a new genus of the difficult-to-culture phylum *Verrucomicrobiota*. Genome analysis, functional annotation, and experimental verification revealed that these two strains degrade polysaccharides and antibiotics, including some complex sulfated polysaccharides (SPs), primarily fucoidan and chondroitin sulfate. Moreover, electron microscopy images revealed that these bacteria can synthesize and store large amounts of glycogen. These polysaccharide degradation and synthesis capacities also exist but differ under nitrogen-deficient conditions, indicating that *Verrucomicrobiota* may have the potential to maintain their normal metabolism by nitrogen fixation under aerobic conditions. Given that polysaccharides and their degradation products are particularly crucial carbon sources for marine microorganisms, *Verrucomicrobiota* are thought to be important contributors to biogeochemical cycling in the ocean.

**IMPORTANCE:**

*Verrucomicrobiota* are widely distributed and able to utilize a variety of difficult-to-biodegrade polysaccharides, which have a significant impact on the marine carbon cycle. However, there are not enough pure culture strains of *Verrucomicrobiota*, as hard-to-cultivate bacteria, for us to study. Here, our study reports a new genus in the phylum *Verrucomicrobiota* and investigates their ability to degrade and synthesize a variety of polysaccharides as well as the mechanism of utilizing difficult-to-degrade polysaccharides. We also explored their special performance on carbon utilization in marine nitrogen-deficient environments. This contributes to deepening our understanding of the involvement of marine microorganisms in the marine carbon cycle.

## INTRODUCTION

The phylum *Verrucomicrobiota* is closely related to the phyla *Planctomycetota* and *Chlamydiota*; together, these phyla form the Planctomycetes–Verrucomicrobiota–Chlamydiae (PVC) superphylum within the bacterial domain. The *Lentisphaerota*, *Elusimicrobiota*, *Desantisiibacteriota*, *Kiritimatiellota*, and *Salta*t*orellota* also belong to the PVC superphylum (1). *Verrucomicrobiota are* widely distributed in many habitats, including oceans, lakes, soils, animals, and the human gut (2–4). However, culturable species remain relatively rare.

*Verrucomicrobiota* play a significant role in the marine carbon cycle (5). These bacteria are able to metabolize a wide range of carbohydrates and polysaccharides, including those that are difficult to degrade. For example, macroalgae-associated *Verrucomicrobiota* use various glycoside hydrolases (HHs), sulfatases, and bacterial microcompartments (BMCs) to degrade sulfated polysaccharides (6). A 2021 report also indicated that members of *Verrucomicrobiota* are associated with diatom blooms and can utilize complex carbohydrates (7). *Verrucomicrobiota* are a potential source of new enzymes, as phylogenetic analysis of β-galactosidase homologs suggests that these proteins form a distinct, putative β-galactosidase subfamily found almost exclusively in *Verrucomicrobiota* (8). In addition, a *Verrucomicrobiota* strain isolated from hot springs in Taiwan’s intertidal zone is reported to contain halophilic thermophilic agar-degrading enzymes (9).

The carbohydrates, particularly polysaccharides, that *Verrucomicrobiota* can degrade constitute an essential part of dissolved organic matter (DOM). The DOM in the Earth’s oceans is comparable in size to the atmospheric carbon dioxide reservoir; it is one of the largest reduced carbon pools on Earth and contains more than 200 times the carbon content of the ocean’s biomass (10). Changes in the oceanic DOM pool are critical to the ocean’s carbon cycle. *Verrucomicrobiota* may influence marine DOM by degrading or synthesizing carbohydrates and polysaccharides, thereby participating in the marine carbon cycle.

However, as a difficult-to-cultivate taxon, only a few strains of *Verrucomicrobiota* have been successfully isolated, and insufficient experimental data restrict our understanding of this phylum. In this study, we report two novel strains of *Verrucomicrobiota*, SDUM461003^T^ and SDUM461004^T^. We examined their basic physiological characteristics and assessed their ability to use carbohydrates and polysaccharides through bioinformatic and experimental methods. This research aims to clarify the role of this *Verrucomicrobiota* group in the DOM and carbon cycles and to provide a new perspective on its ecological significance.

## RESULTS

### Isolation and identification of strains SDUM461003^T^ and SDUM461004^T^

**Fig 1:**
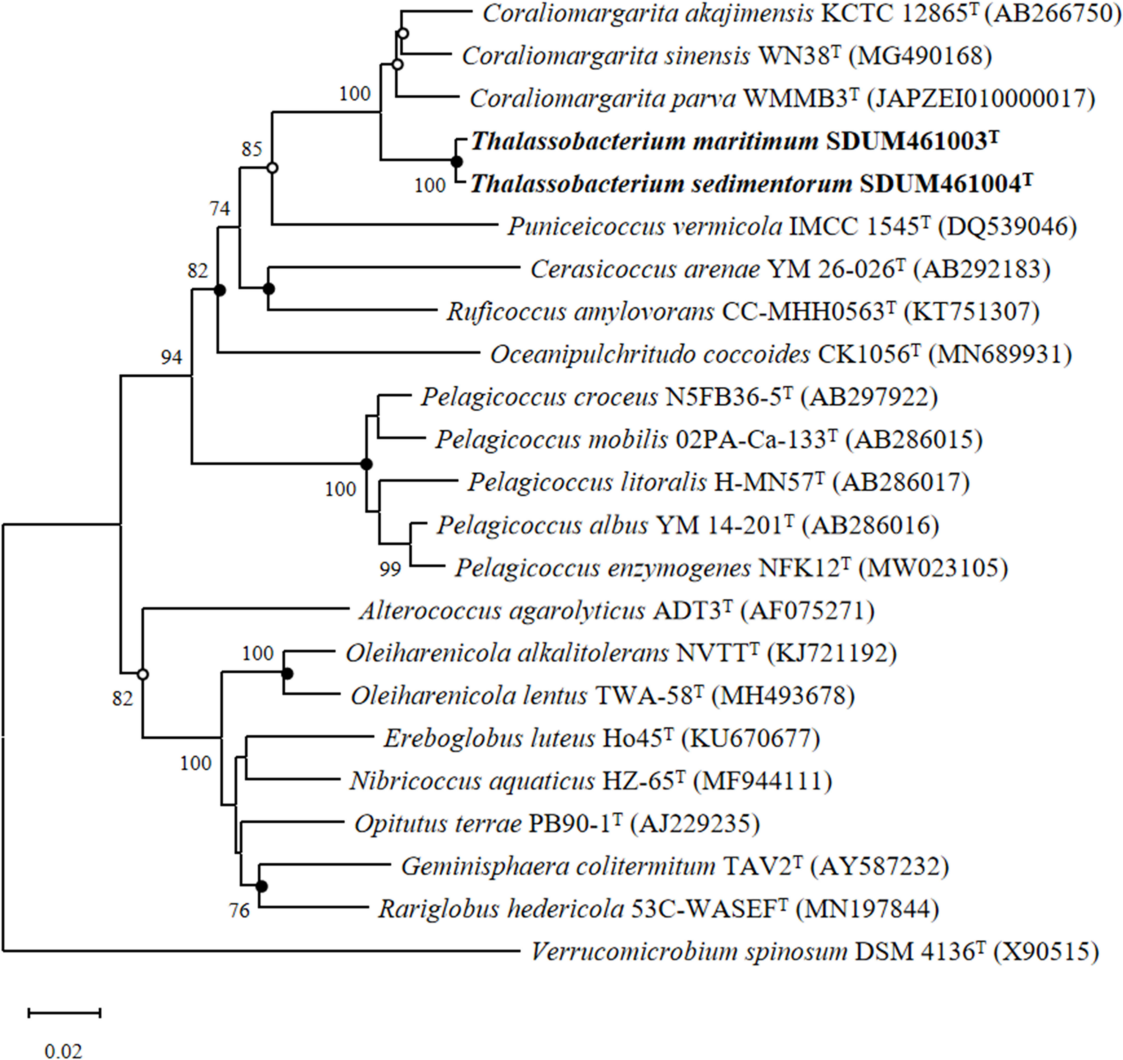
Neighbor-joining phylogenetic tree based on the complete 16S rRNA gene sequences showing the positions of strains SDUM461003^T^ and SDUM461004^T^. Bootstrap values (>70%) are shown at branch nodes. *Verrucomicrobium spinosum* DSM 4136^T^ was used as the root.

SDUM461003^T^ and SDUM461004^T^ were isolated from marine sediments in Weihai, China (36°58′37″ N, 122°2′37″ E). The cells of both strains are spherical in shape. The diameter of SDUM461003^T^ cells is approximately 0.45–0.65 µm, while that of strain SDUM461004^T^ is approximately 0.40–0.75 µm (Fig. S1 represents SDUM461003^T^, and b and d represent SDUM461004^T^). Both strains are reproduced by binary fission (Fig. S1). Neither strain exhibits motility. The nearly complete 16S rRNA gene sequences of SDUM461003^T^ (1603 bp) and SDUM461004^T^ (1529 bp) were obtained and compared to those of related bacterial species available in the EzBioCloud database. The results revealed that the two strains are most similar to *Coraliomargarita sinensis* WN38^T^ (Table S1), with ANI values of 73.5% and 73.1% and AAI values of 71.6% and 71.5%, respectively. Phylogenetic trees were constructed based on 16S rRNA gene sequences using neighbor-joining, maximum likelihood, and maximum parsimony methods. The results revealed that these two strains establish a clade next to the genus *Coraliomargarita* (Fig. 1). Additionally, AAI and ANI values were used to determine the evolutionary relationships of strains SDUM461003^T^, SDUM461004^T^, *C. sinensis* WN38^T^, *C. akajimensis* KCTC 12865^T^, and *C. parva* WMMB3^T^ (Table S1). The AAI value among these five strains was less than 72%, and the highest ANI value between any two strains was 82.6% (SDUM461003^T^ and SDUM461004^T^). The fact that the AAI and ANI values are below the thresholds for genus and species delineation (AAI 65–72% and ANI < 95%) indicates that SDUM461003^T^ and SDUM461004^T^ represent two distinct novel species within a new genus.

To further explore their characteristics, the genomes of SDUM461003^T^ and SDUM461004^T^ were sequenced and analyzed. The genomic data of strains *C. sinensis* WN38T, *C. akajimensis* KCTC 12865^T^, and *C. parva* WMMB3^T^ were obtained from the NCBI database (Table S2). The genome of SDUM461003T contains 4,092 genes, 46 tRNAs, 3 rRNAs, and 4 ncRNAs. In comparison, SDUM461004^T^ has 3,778 genes, 40 tRNAs, 3 rRNAs, and 4 ncRNAs. The DNA G + C content of the genus *Coraliomargarita* was 53.9–56.0%, while the DNA G + C contents of strains SDUM461003^T^ and SDUM461004^T^ were 52.2% and 50.3%, respectively.

### Metabolic pathway analysis of two novel strains and *Coraliomargarita* strains

**Fig 2:**
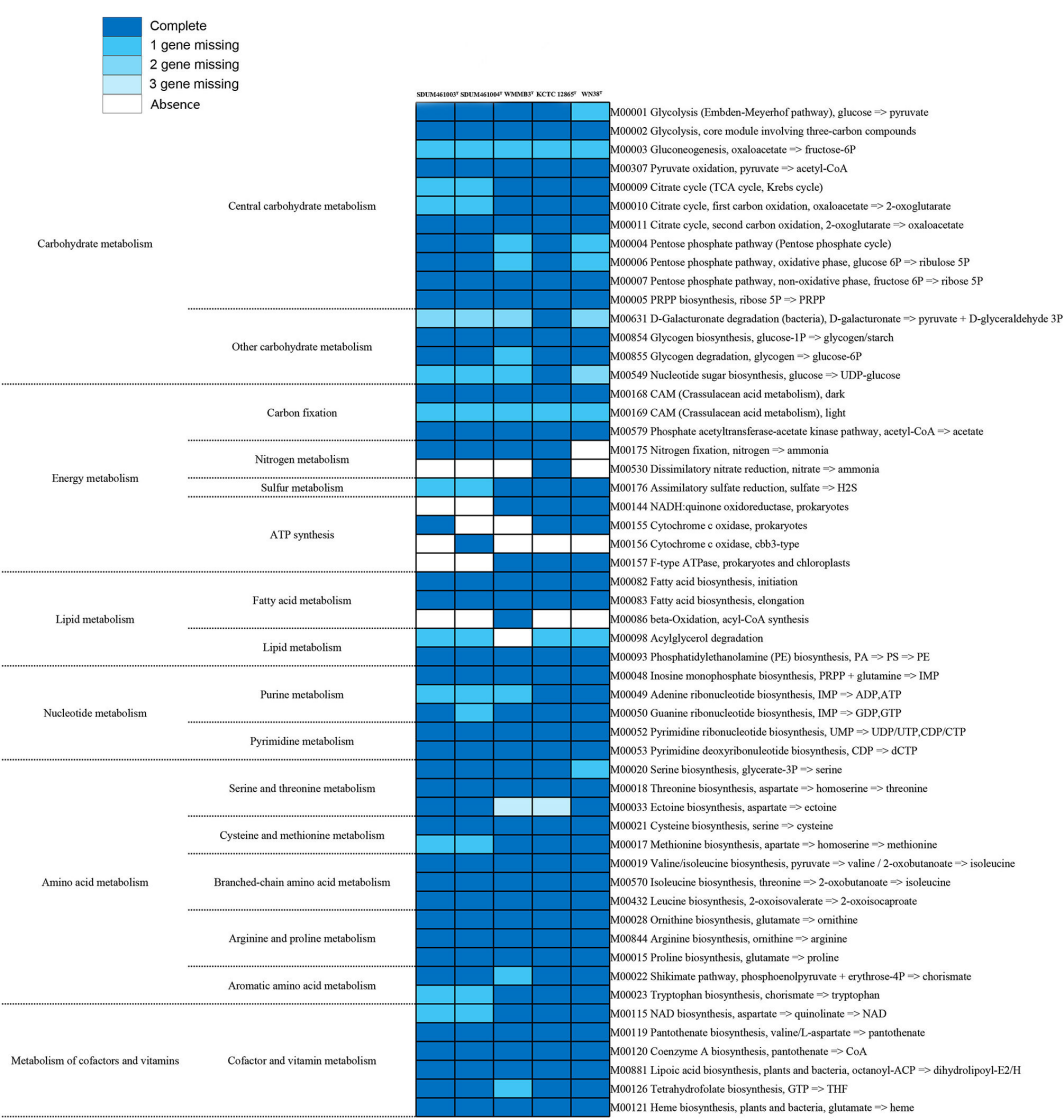
Metabolic pathways identified through KEGG in the genus *Coraliomargarita*, strain SDUM461003^T^, and strain SDUM461004^T^. The colors represent the number of genes missing.

The two novel strains appear to be most closely related to *C. parva* WMMB3^T^, *C. sinensis* WN38^T^, and *C. akajimensis* KCTC 12865^T^. Therefore, these three strains were selected for metabolic pathway analysis and comparison. We found that they had multiple genes that encode proteins associated with carbohydrate metabolism, energy metabolism, lipid metabolism, nucleotide metabolism, amino acid metabolism, and the metabolism of cofactors and vitamins (Fig. 2).

Our analysis of the genomes of the strains SDUM461003^T^ and SDUM461004^T^ indicates that these strains are able to produce the enzymes necessary to catalyze several complete metabolic pathways of carbohydrate metabolism, including glycolysis (M00002), pyruvate oxidation (M00307), the citrate cycle (M00011), the pentose phosphate pathway (M00007), and PRPP (phosphoribosyl pyrophosphate) biosynthesis (M00005). They lack a gene that encodes ICDH (isocitrate dehydrogenase, EC:1.1.1.42) in the citrate cycle (M00009 and M00010). This is a key enzyme in the TCA cycle. With respect to energy metabolism, they possess the complete crassulacean acid metabolism (CAM, dark) (M00168) and the phosphate acetyltransferase-acetate kinase pathway (M00579), but the crassulacean acid metabolism (CAM, light) (M00169) pathway is incomplete due to the absence of MaeB (malate dehydrogenase, EC:1.1.1.40). In addition, both strains also have a complete nitrogen fixation pathway (M00175) through which they can convert nitrogen into ammonia. Each strain lacks a gene in the assimilatory sulfate reduction pathway (M00176). With respect to lipid metabolism, both strains possess all the genes necessary for complete fatty acid biosynthesis (M00082 and M00083) and phosphatidylethanolamine (PE) biosynthesis (M00093), and PE was identified among the polar lipids of these strains (Fig. S2). The strains lack complete pathways for adenine ribonucleotide biosynthesis metabolism (M00049), methionine biosynthesis (M00017), tryptophan biosynthesis (M00023), and NAD biosynthesis (M00115).

Unlike the *Thalassobacterium* strains SDUM461003^T^ and SDUM461004^T^, the *C. akajimensis* strain KCTC 12865^T^ possesses a complete dissimilatory nitrate reduction pathway (M00530), providing a competitive advantage in nitrogen-limited environments, a characteristic that is significant for the global nitrogen cycle. The assimilatory sulfate reduction pathway (M00176) is complete in the *Coraliomargarita* strains, suggesting that those strains also play an important role in the sulfur cycle. The close relationship between strains SDUM461003^T^ and SDUM461004^T^ compared to that of the *Coraliomargarita* strains is evident.

### Analysis of carbon source utilization

#### The capacity to utilize simple small organic molecules

The ability of SDUM461003^T^ and SDUM461004^T^ to utilize various carbon sources was tested using BIOLOG GEN III MicroPlates. The positive results are shown in Table S3. These bacteria are able to utilize many different types of monosaccharides and small-molecule oligosaccharides. Their ability to utilize a wide range of carbon sources demonstrates their ecological role in the interaction between DOM and marine microbes and indicates that they are potentially involved in the process of marine carbon cycling through many pathways.

In addition to conventional carbon sources, the two novel strains are also able to use rifamycin and vancomycin as carbon sources; this is another indication that they can affect the marine DOM pool. These antibiotics are secreted by marine bacteria as a means of inhibiting competitors (11, 12). The ability of SDUM461003^T^ and SDUM461004^T^ to utilize these antibiotics may help them defend against threats from other bacteria in the environment. To confirm this survival strategy, we also conducted antibiotic resistance experiments.

**Fig 3:**
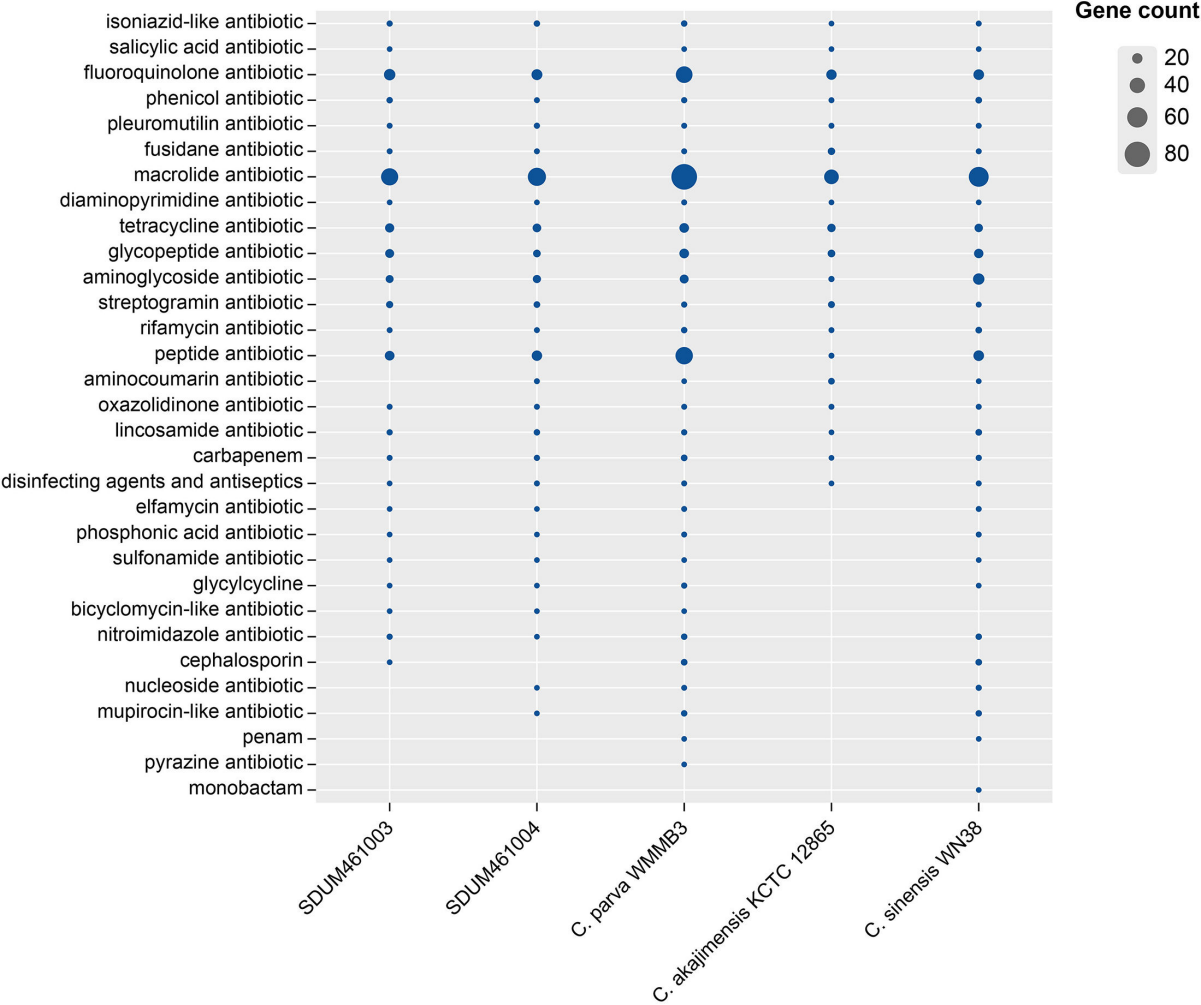
Analysis of antibiotic resistance genes between strains SDUM461003^T^, SDUM461004^T^, and *Coraliomargarita*. The sizes of the bubbles represent the numbers of gene clusters.

The antibiotic resistance genes were annotated using the CARD database (Fig. 3), and the antibiotic resistance of strains SDUM461003^T^ and SDUM461004^T^ was experimentally confirmed (Table S4). The genomes were annotated in the CARD database using both strict and loose algorithms. Strains SDUM461003^T^ and SDUM461004^T^ are remarkably resistant to macrolide antibiotics and fluoroquinolone antibiotics. The experimental results confirmed that both strains are resistant to macrolide antibiotics (erythromycin) and that SDUM461003^T^ is resistant to fluoroquinolone antibiotics (norfloxacin). Genome annotations indicate that the two strains carry genes associated with resistance to glycopeptide antibiotics and rifamycin. Notably, despite having relatively few genes that encode proteins that specifically target vancomycin and rifamycin, SDUM461003^T^ and SDUM461004^T^ present phenotypes associated with both antibiotics.

The BIOLOG results revealed that these strains degrade multiple small-molecule carbon sources. Combined with the antibiotic resistance data, it can be speculated that their vancomycin and rifamycin resistance may be linked to metabolic activity. These findings suggest that when comprehensively assessing the microorganisms’ contribution to the carbon cycle, we must fully consider the wide-ranging survival environments that they might encounter so that we can precisely understand the intricate and changing relationship between microorganisms and the carbon cycle. It is essential to conduct research not only on the metabolic capabilities of microorganisms under normal conditions but also on how they adapt to different environments and interact with them when confronted with various stresses.

### The capacity to utilize macromolecular complex polysaccharides

The potential gene clusters associated with polysaccharide degradation in the genomes of five strains (strains SDUM461003^T^, SDUM461004^T^, *C. sinensis* WN38^T^, *C. akajimensis* KCTC 12865^T^, and *C. parva* WMMB3^T^) were annotated using the methods of Lu et al. (13). These gene clusters were categorized into PUL (polysaccharide utilization loci), PUL-like, and CGCs (CAZyme gene clusters) based on the presence or absence of SusCD and TonB. PULs, which contain SusCD genes, are common types of polysaccharide degradation gene clusters found in the genomes of *Bacteroidetes*. In contrast, SusCD was not found in the annotations of any of the five *Verrucomicrobiota* strains in this study. However, all five strains possess the TonB system. The type of gene cluster containing the TonB system is referred to as PUL-like. Gene clusters that contain neither SusCD nor TonB but contain only genes encoding the glycoside hydrolase (GH) family, the glycosyltransferase (GT) family, the polysaccharide lyase (PL) family, the carbohydrate esterase (CE) family, the auxiliary activity (AA) family, and carbohydrate-binding modules (CBMs) are called CGCs.

**Fig 4:**
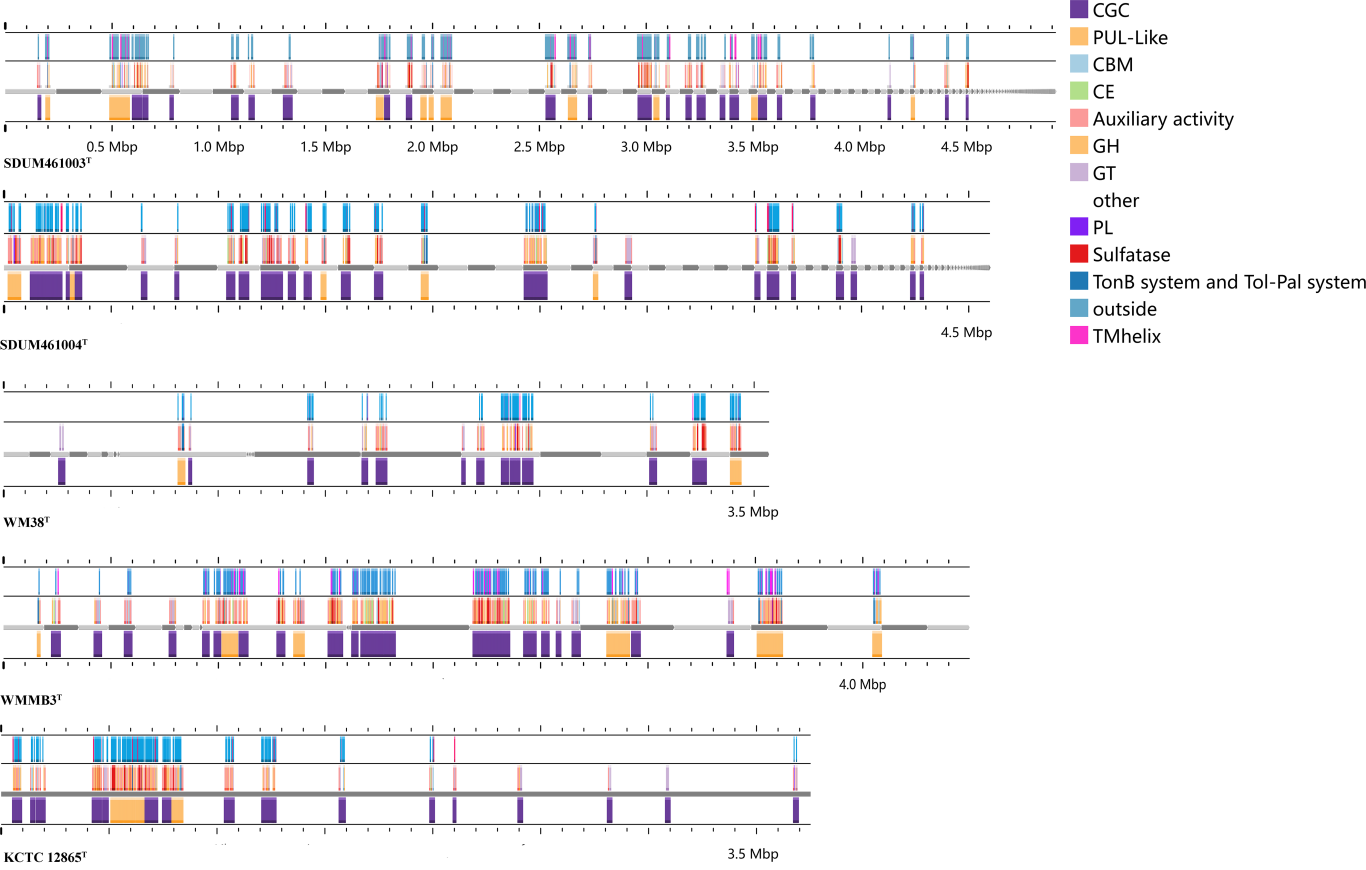
Composition and location of PUL-like clusters and CGCs in the genomes of different strains. The strain names are shown below each graphic. The bands from bottom to top indicate the type of polysaccharide degradation gene cluster, the composition of the gene cluster, and the distribution location of its protein components.

We compared the PUL-like and CGC genes annotated in different strains. The gene clusters annotated in the genomes of strains SDUM461003^T^ and SDUM461004^T^ and in the genus *Coraliomargarita* are shown in Fig. 4 and Fig. S3. Among the examined strains, the greatest numbers of PUL-like clusters and CGCs were annotated in SDUM461003^T^ and SDUM461004^T^. All the strains were annotated to have at least two PUL-like clusters and 12 CGCs (Fig. 4). These findings indicate that these strains have great potential to degrade complex polysaccharides. A comparison of the compositions and positions of the gene clusters (Fig. 4) revealed that even within such a small taxonomic branch, the composition of the PUL-like and CGC gene clusters was highly variable. These findings demonstrate the variety of polysaccharides utilized by strains within this taxon and the complexity of the mechanisms involved (6). By performing signal peptide prediction of PUL genes, we found that most of these genes encode proteins that are secreted outside of the membrane rather than being anchored to the cell membrane or retained inside the cell. By secreting metabolic substances and degrading external carbohydrates, these strains may impact microbial communities in the surrounding environment.

**Fig 5:**
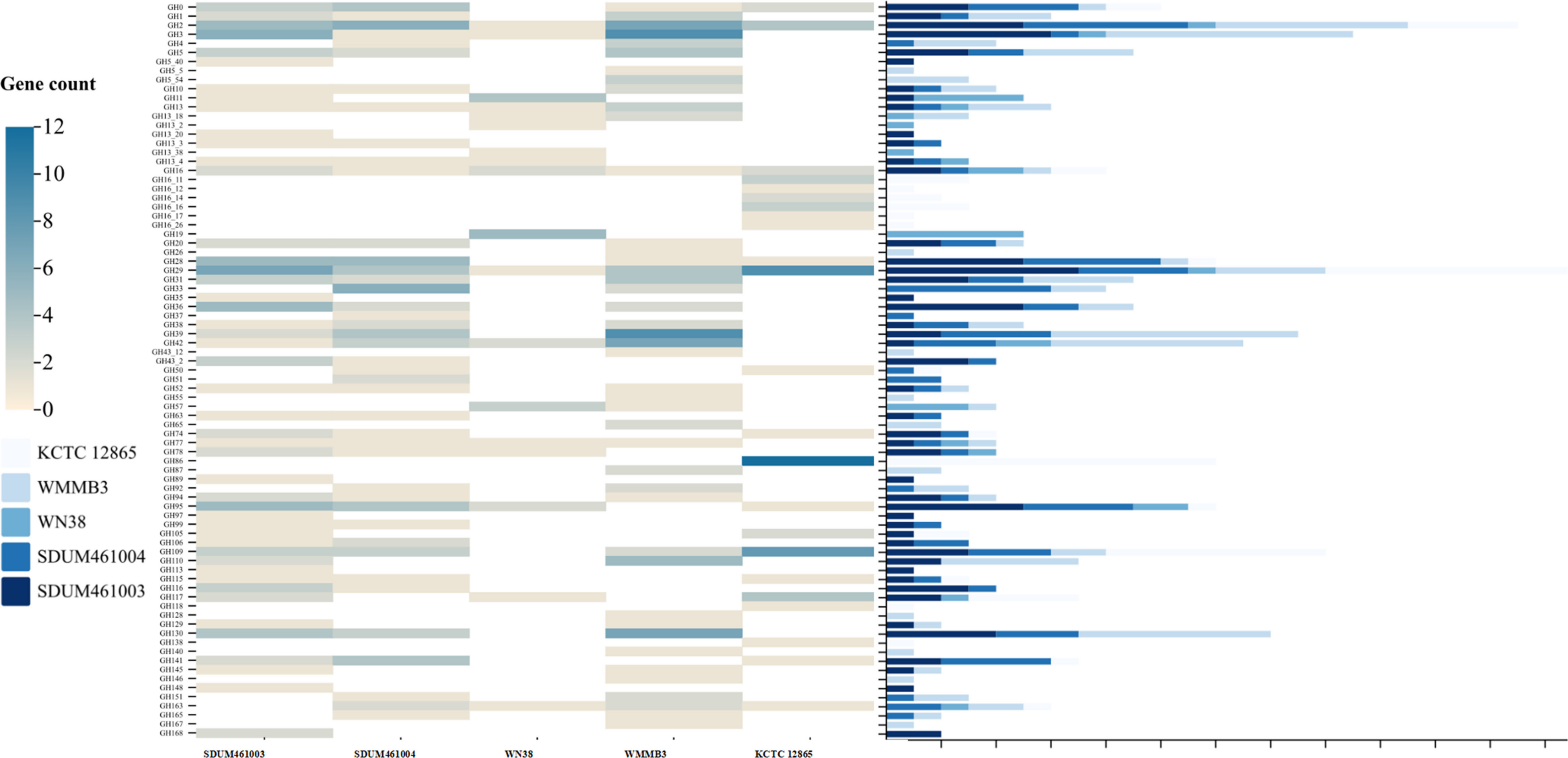
The panel on the left shows the GH gene content in PUL-like clusters and CGCs in different strains. The panel on the right shows the number of GH gene superpositions corresponding to all strains in this classification.

For further study, we focused on the GH family found in PUL-like clusters and CGCs (Fig. 5). Among the examined strains, the gene with the greatest number of gene copies was GH86, which encodes β-agarase (EC 3.2.1.81) that cleaves the β-1,4 glycosidic bonds of agarose. However, GH86 was present only in *C. akajimensis* KCTC 12865^T^ and was not found in any of the remaining four strains. This suggests that, of the strains present in this taxon, *C. akajimensis* KCTC 12865^T^ may have a unique and potentially powerful agar degradation capability. GH2 and GH29 were widely present in all strains, and the numbers of these genes were also significantly greater than the other GH family genes. GH2 encodes galactosidase (EC 3.2.1.23), which hydrolyzes ɑ-L-arabinosides, and mannosidase (EC 3.2.1.25), which hydrolyzes terminal nonreducing β-D-mannose residues in β-D-mannosides, along with other enzymes. GH29 encodes α-L-fucosidase (EC 3.2.1.51), α-1,3/1,4-L-fucosidase (EC 3.2.1.111), which hydrolyzes (1→3)-linkages between α-L-fucose and N-acetylglucosamine residues in glycoproteins, along with other enzymes. Among the gene clusters identified in these strains, the most common were clusters of sulfatase-encoding genes. Such genes encode proteins that function in the hydrolysis of glycosidic bonds. Since fucose is the primary component of fucoidan, these results further suggest that strains within this taxon are capable of degrading sulfated polysaccharides (SPs). Indeed, there is increasing evidence that some *Verrucomicrobiota* can degrade SPs (7). It has been reported that algae contain many SPs in their cell walls and extracellular matrix, and this helps them survive under harsh seawater conditions (14). Strains SDUM461003^T^ and SDUM461004^T^, which belong to the family *Coraliomargaritaceae*, are found on the surface of kelp and can reach an abundance of up to 6.7% during kelp decay (5). *Verrucomicrobiota* maintain a symbiotic relationship with some plants (15, 16). Their ability to degrade polysaccharides and their preference for degrading SPs may be key to maintaining their relationship with kelp. DOM is also an important component of the interaction between marine microorganisms and the DOM pool. The experimental results revealed that the examined strains are able to utilize large amounts of difficult-to-degrade molecules and insoluble polysaccharides, an ability that may have a strong influence on DOM and fixed carbon.

**Fig 6:**
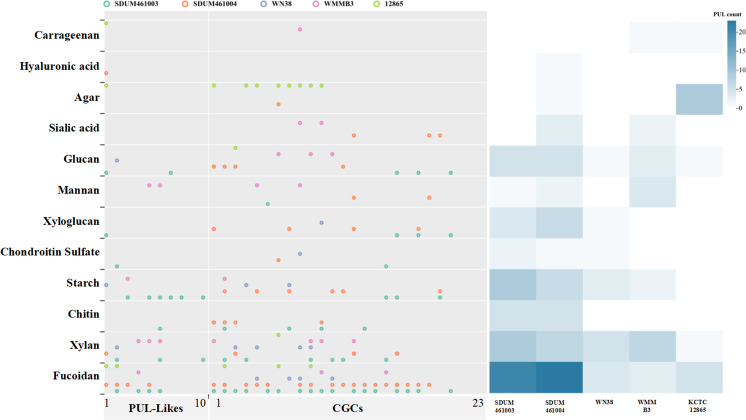
Specific polysaccharides that can be degraded due to the presence of each PUL-like cluster or CGC. The panel on the right indicates the number of gene clusters through which each strain can degrade specific polysaccharides.

The polysaccharides that can be degraded by the strains were analyzed based on gene clusters. Strain SDUM461003^T^ has the potential ability to degrade fucoidan, xylan, chitin, starch, chondroitin sulfate, xyloglucan, mannan, and dextran. Strain SDUM461004^T^ can also degrade salivary acid and hyaluronic acid. The remaining results are shown in Fig. 6. The results predict that *Thalassobacterium* can generally degrade complex polysaccharides such as fucoidan, xylan, glucan, starch, and other complex polysaccharides. Individual strains have carrageenan- and agar-utilizing abilities.

**Fig 7:**
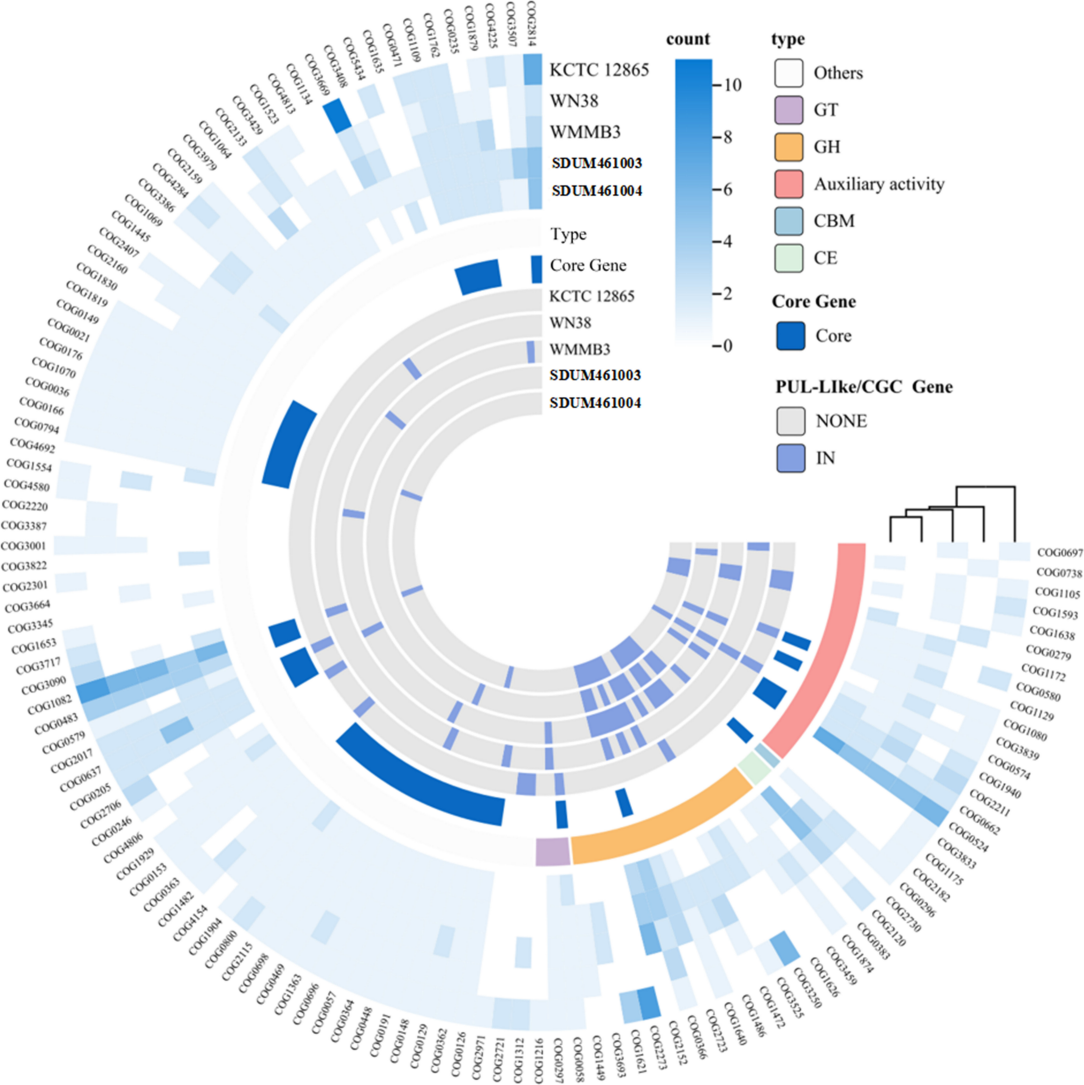
Pangenomic annotation of the genus *Coraliomargarita*, strain SDUM461003^T^, and strain SDUM461004^T^.

To validate the distributions of the gene clusters found in the five strains (SDUM461003^T^, SDUM461004^T^, *C. sinensis* WN38^T^, *C. akajimensis* KCTC 12865^T^, and *C. parva* WMMB3^T^), we performed pangenomic annotation. The results are shown in Fig. 7. We focused on genes associated with polysaccharide degradation in the COG classification. Among the polysaccharide degradation genes of these bacteria, only a small fraction of the genes that were annotated as PUL-like or CGCs were core genes, further illustrating the diversity of polysaccharide degradation mechanisms. Many of the core genes associated with polysaccharide degradation were not identified during the previous annotation process. The findings indicate that these strains have great potential to utilize polysaccharides in several novel ways. Thus, attention was given to those hydrolases that were annotated in multiple strains but not in PUL-like strains or CGCs. It was found that the substrates that *Thalassobacterium* prefers are alginate (COG4813) and lignin (COG2159) but not fucose.

**Fig 8:**
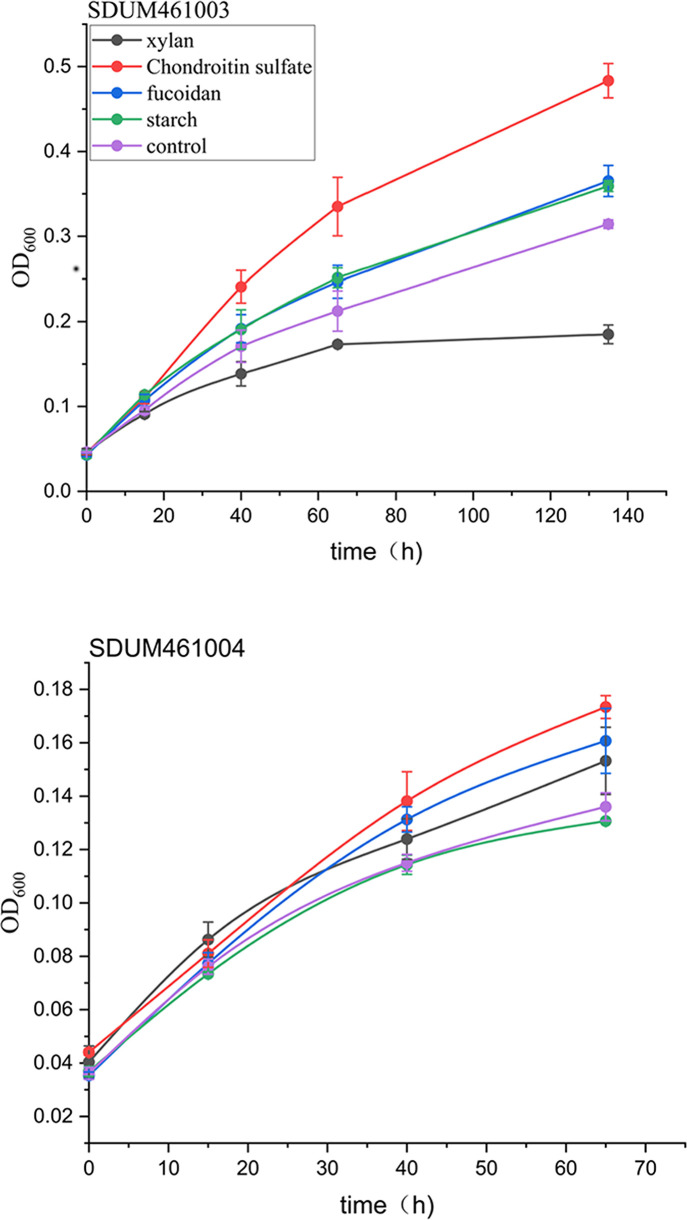
OD_600_ values of the strains after growth in the presence of various polysaccharides (xylan, chondroitin sulfate, fucoidan, and starch).

To further validate the results of the genomic analysis, biochemical experiments were performed using SDUM461004^T^ and SDUM461003^T^. The results showed that SDUM461003^T^ is able to utilize starch, chondroitin sulfate, and fucoidan (Fig. 8). Of these, chondroitin sulfate has the strongest growth-promoting effect. SDUM461004^T^ is able to utilize xylan, fucoidan, and chondroitin sulfate. Sulfated polysaccharides (chondroitin sulfate and fucoidan) both have strong ability to promote growth (Fig. 8).

In summary, we predicted the polysaccharide utilization abilities of five bacterial strains from the evolutionary branch containing SDUM461004^T^ and SDUM461003^T^. We analyzed the compositions of the polysaccharide degradation gene clusters and their genomic localizations and explored the extracellular secretion of polysaccharide-degrading enzymes. The PULs were found to be very different from the PULs of other bacteria that utilize polysaccharides. The examined strains do not contain SusCD genes that confer the ability to transport polysaccharides. Instead, they mainly transport them through the TonB system. Their genomes encode numerous sulfate esterases, suggesting that their ability to degrade SPs is strong. This rare ability may be related to their symbiotic relationships with some marine plants, and it has great application potential. The results of the pangenomic analysis and the validation experiments showed that the polysaccharide degradation gene clusters found in the species on this branch are diverse and suggest that unknown mechanisms with great potential for the utilization of complex polysaccharides may exist. Dissecting these unknown mechanisms is highly important for understanding the interactions of microbes in the marine environment with DOM and the ocean carbon cycle.

### Analysis of the abilities of the strains to synthesize polysaccharides

**Fig 9:**
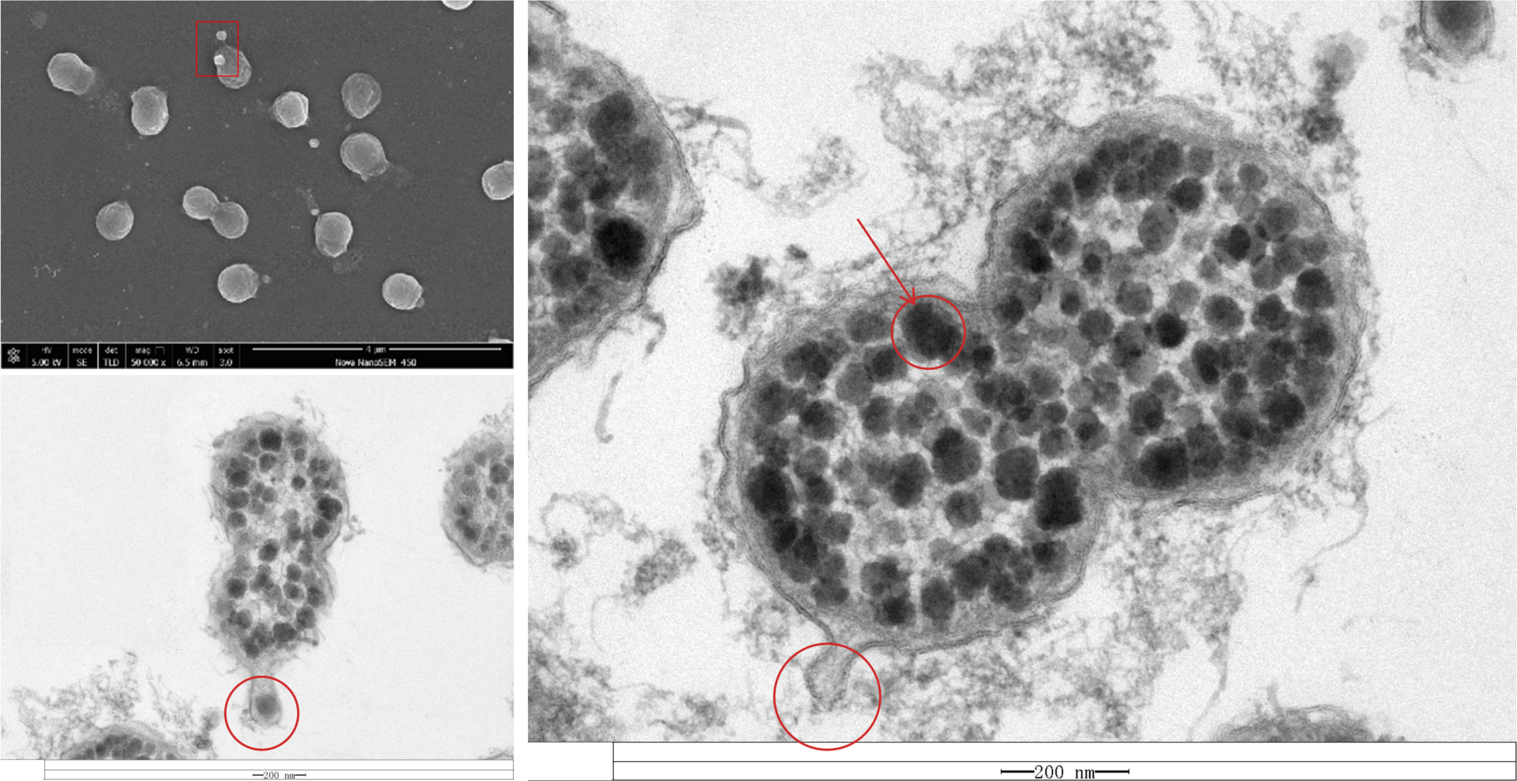
Scanning electron and transmission electron micrographs of SDUM461003^T^. The transmission electron micrographs were obtained from adjacent fields of view in a single imaging session. The arrow points to glycogen, and the circled portion of the images shows the outer membrane vesicles (OMVs).

In the scanning electron microscopy images of SDUM461003^T^, small vesicles were observed near the cells (Fig. S1). For further observation, we obtained transmission electron micrograph (TEM) images (Fig. 9). In the TEM images, the cells are surrounded by a layer of material that is presumed to be part of the biofilm matrix. The cytoplasm is coated with numerous black particles, which are assumed to be glycogen based on previous reports (17). Additionally, based on previous results, the cells secrete vesicles (most of the PUL genes encode proteins that are secreted toward the outside of the membrane). We hypothesize that the cells secrete CAZymes via vesicles (Fig. 9; vesicles are marked with red circles). This behavior has also been reported in previous studies (18). The secretion of CAZymes results in the degradation of macromolecular polysaccharides in the extracellular environment and may affect the surrounding biological community; thus, it has ecological significance.

Glycogens, which are known to serve as a form of energy and carbon storage, are part of a mechanism by which bacteria adapt to oligotrophic marine environments. According to metabolic analyses, all of the strains examined in this study had an intact glycogen synthesis pathway, further supporting the likelihood that the black particles observed in the cytoplasm consist of glycogen. Moreover, these strains belong to a group that is not classified in the same way as was previously reported. Therefore, it may be inferred that the capacity to use glycogen for carbon storage is common among *Verrucomicrobiota*. The observations suggest that strain SDUM461003^T^ is capable of degrading a wide range of sulfated complex polysaccharides and of converting polysaccharides to glycogen.

### Polysaccharide utilization and polysaccharide synthesis under conditions of external nitrogen deficiency

**Fig 10:**
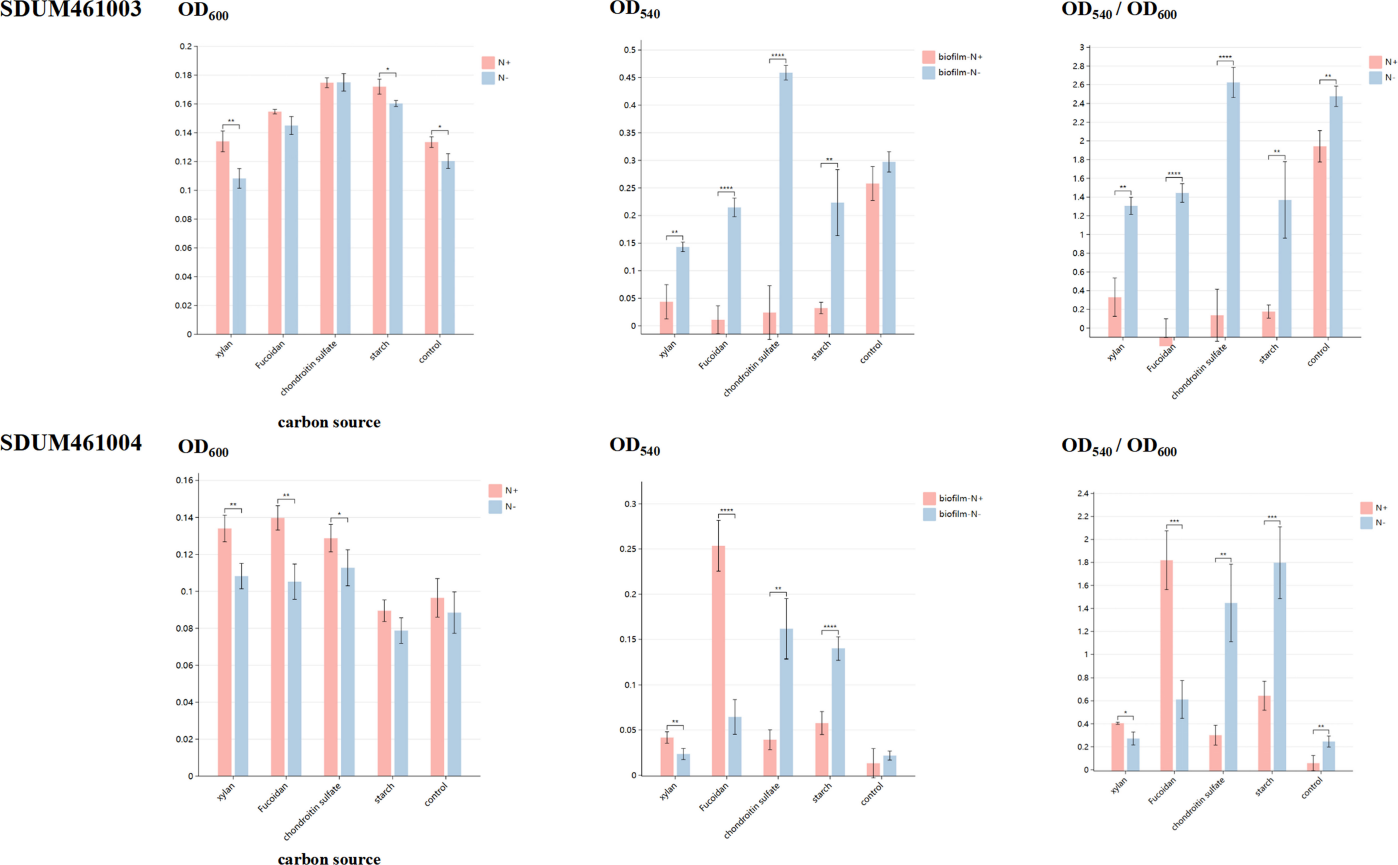
Growth and biofilm volumes of the two strains after culture in nitrogen-containing and nitrogen-free environments in medium supplemented with various polysaccharides. The vertical coordinates show the values after the blank group was subtracted, and the horizontal coordinates show the carbon sources that were added. “*” indicates the degree of statistical significance. Data points not labeled “*” are not significantly different from each other.

Strains SDUM461004^T^ and SDUM461003^T^ were isolated from marine environments. Considering that the ocean is a nitrogen-limited environment, exploring the ability of strains to degrade and synthesize polysaccharides and thereby affect DOM in the ocean should not be considered only in terms of nutrient-sufficient conditions. We compared the polysaccharide utilization and biofilm synthesis capacity of the two strains in nitrogen-containing and nitrogen-free media. The OD_600_ values were measured for cultures grown under conditions of nitrogen restriction or nitrogen addition in the presence of various polysaccharides. The ability of the strains to utilize different polysaccharides was evaluated based on the OD_600_ values of the cultures. The amount of biofilm formed under the tested conditions was also measured; this was done with crystal violet staining, and the results are expressed as OD_540_. The results of these experiments are shown in Fig. 10. The OD values represent the values that were obtained after the values for the blank groups were subtracted; statistical significance is indicated by asterisks (*P < 0.05; **P < 0.01; ***P < 0.001; ****P < 0.0001). Polysaccharides are the main components of biofilms (19), and the biofilms wrapped around bacteria not only serve as polysaccharides but also participate in the carbon cycle by contributing to the aggregation and deposition of carbon particles, a process that promotes the formation of carbon sinks (20). Notably, the strains were able to utilize polysaccharides and grow in nitrogen-free medium in an aerobic environment. Although the metabolic analysis showed that both strains contained the nif gene, nitrogen fixation generally occurs only under anaerobic conditions (21), and although strains capable of aerobic nitrogen fixation do exist (22), they have not yet been reported among *Verrucomicrobiota* or even among species that exist in marine environments. The two novel strains, SDUM461004^T^ and SDUM461003^T^, are able to fix nitrogen under aerobic conditions, allowing them to achieve normal polysaccharide utilization and synthesis. This is an important discovery that furthers our understanding of the ecological characteristics of *Verrucomicrobiota*.

When we compared the growth of strains in media containing various polysaccharides under nitrogen-addition and nitrogen-restriction conditions, we found that under both conditions, SDUM416003^T^ was able to utilize fucoidan, chondroitin sulfate, and starch and that SDUM416004^T^ was able to utilize xylan, fucoidan, and chondroitin sulfate. This is consistent with the results of the carbon sources analysis described in the previous sections. Growth in a medium containing the same polysaccharide under nitrogen-addition and nitrogen-free conditions was compared. Under these two conditions, SDUM461003^T^ showed no significant difference in growth with fucoidan or chondroitin sulfate, while the growth of SDUM461003^T^ was poorer under nitrogen-free conditions. There was no significant difference in the growth of SDUM461004^T^ between the control group and the starch-addition group, but its growth was greater under nitrogen-addition conditions, similar to that of SDUM461003^T^. This result is predictable since bacteria need both carbon and nitrogen sources to grow, and reduction of the nutrient content affects their growth. However, the results show that there was relatively little difference in the growth of the strains under nitrogen-addition and nitrogen-free conditions; the largest difference was approximately 24%, and it occurred under nitrogen-addition growth conditions in the SDUM461004^T^ fucoidan-addition group. Although there was a downward trend, there was no statistically significant difference in some experimental groups (the SDUM461003^T^ fucoidan-addition and chondroitin sulfate-addition groups and the SDUM461004^T^ starch-addition and control groups). Statistical significance is indicated by * in Fig. 10. This suggests that even in the ocean, which constitutes a nitrogen-deficient environment, the ability of these two bacterial strains to use polysaccharides is not greatly reduced, possibly because they are able to obtain enough nitrogen through nitrogen fixation to maintain their growth.

To assess the impact of biomass on biofilms, biofilm production per unit of biomass was expressed as OD_540_/OD_600_. The amount of biofilm produced in different media differed significantly in both the presence and absence of nitrogen. In the control group without polysaccharide, both SDUM461003^T^ and SDUM461004^T^ produced significantly greater amounts of biofilm under nitrogen-free conditions than under nitrogen-containing conditions. When grown in the presence of polysaccharides, SDUM461003^T^ produced significantly more biofilm under nitrogen-free conditions than under nitrogen-containing conditions, just as for the control in the absence of polysaccharide. SDUM416004^T^ produced more biofilm when grown with xylan or fucoidan in the presence of nitrogen and when grown with chondroitin sulfate or starch in the absence of nitrogen. It also produced less biofilm per unit in the experimental group than in the control group, except when grown in the presence of chondroitin under nitrogen-free conditions. In contrast, the amount of biofilm produced by SDUM461003^T^ in the experimental groups was greater than that in the control group. In previous studies of aerobic nitrogen fixation, it has been suggested that biofilm synthesis is a way in which bacteria protect nitrogen-fixing enzymes from oxygen toxicity (23). Considering that the nitrogen fixation of strains SDUM461003^T^ and SDUM461004^T^ occurred under aerobic conditions, the increase in the amount of biofilm formed under nitrogen-deficient conditions could be due to this. Bacteria promote biofilm synthesis to achieve nitrogen fixation under aerobic conditions to survive in nitrogen-deficient environments. This process is also related to the metabolism of carbon sources in the habitat. In addition to the changes that were observed under nitrogen-addition and nitrogen-free conditions, the abilities of the two strains to synthesize biofilms in the presence of different polysaccharides were also enhanced and weakened to different degrees. The generation of biofilms by bacteria affects the marine carbon sink and the DOM reservoir. Understanding the differences in the metabolism of bacteria in different growth environments can help us better understand the marine carbon cycle.

In summary, these results show that SDUM461003^T^ and SDUM461004^T^ respond differently to different polysaccharides. Their growth and the amount of biofilm they synthesized differed depending on the polysaccharides that were present, as did the effects of nitrogen-limiting conditions. In marine nitrogen-limited environments, they had reduced biomass but exhibited increased biofilm synthesis, except in the case of SDUM461004^T^ under conditions in which xylan and fucoidan were added. This mechanism may allow strains to participate in the carbon cycle, helping carbon particles settle and contributing to carbon sinks.

### Chemotaxonomic analyses

In strain SDUM461003^T^, the major fatty acids (>5%) were C_16:0_ (7.5%), C_18:0_ (15.1%), iso-C_14:0_ (15.2%), anteiso-C_15:0_ (6.7%), and C_18:1_ ω9c (27.1%) (Table S5). The major polar lipids were phosphatidylglycerol (PG), diphosphatidylglycerol (DPG), phosphatidylethanolamine (PE), aminophospholipid (APL), and three unidentified lipids (L) (Fig. S2). In strain SDUM461004^T^, the major fatty acids (>5%) were C_14:0_ (11.1%), C_16:0_ (10.5%), C_18:0_ (11.0%), iso-C_14:0_ (14.9%), anteiso-C_15:0_ (6.3%), and C_18:1_ ω9c (25.5%) (Table S5). The major polar lipids present in SDUM461003^T^ were DPG, PE, and four unidentified lipids (L) (Fig. S2). Strains SDUM461003^T^, SDUM461004^T^, and others had several main fatty acids (C_18:0_, iso-C_14:0_, and C_18:1_ ω9c) in common. The main respiratory quinone present in strains SDUM461003^T^ and SDUM461004^T^ was methyl naphthoquinone (MK-7).

## DISCUSSION

In this study, two novel strains of *Verrucomicrobiota* were isolated; these strains constitute a new genus, *Thalassobacterium*. We focused on exploring the polysaccharide degradation and synthesis capabilities of this new genus, as well as the changes observed under nitrogen-limited conditions. The aim of this study was to use carbon metabolism characteristics as a starting point to investigate how *Verrucomicrobiota* affect the marine DOM pool and how they participate in the carbon cycle in the marine environment.

First, through genomic annotation and verification, we found that *Thalassobacterium* has the ability to utilize complex macromolecular polysaccharides such as fucoidan, xylan, glucan, starch, and agar. Its PUL-like gene clusters contain genes that encode multiple sulfatases that help degrade SPs. *Verrucomicrobiota* have been widely reported to have the ability to degrade SPs (6), but the mechanism through which they degrade these compounds is particularly intriguing. Through gene annotation and experiments conducted on these strains, we were able to confirm and explore this degradation mechanism. Notably, the PUL-like gene clusters identified in this study lack SusCD genes but include genes encoding components of the TonB system, with most CAZymes being secreted extracellularly. This suggests two hypotheses: first, the strains may be able to transport polysaccharides into the cell via the TonB system; second, the strains may secrete CAZymes extracellularly to degrade polysaccharides outside the cell. The latter has also been reported in other bacteria (18). We believe that the two mechanisms likely coexist. The second hypothesis is partially supported by the TEM images, which revealed numerous vesicles. These vesicles are likely involved in the extracellular secretion of CAZymes. If correct, this hypothesis would suggest that *Thalassobacterium* not only utilizes macromolecular polysaccharides as carbon sources but also degrades these polysaccharides extracellularly, thereby helping other microbes in the environment access degradable small-molecule carbon sources more easily. This would significantly amplify its impact on the marine DOM pool and, if it occurs, would indicate that *Thalassobacterium* plays an important and complex role in the marine carbon cycle. However, this hypothesis has not yet been experimentally verified. In future research, we plan to focus on the vesicles produced by these strains, isolating them and identifying the enzymes they contain. Such studies will help deepen our understanding of the polysaccharide degradation mechanisms employed by *Verrucomicrobiota* and will provide valuable insights into the role of microbes in the marine carbon cycle.

Second, the work reported here provides evidence that *Thalassobacterium* influences the DOM pool through the synthesis of large amounts of polysaccharides. The TEM images we obtained show that the strains store large amounts of glycogen intracellularly and that they produce extracellular polysaccharides (EPSs) that form biofilms. Our findings suggest that *Thalassobacterium* converts polysaccharides that are difficult to degrade into the more easily degradable compound glycogen. Glycogen storage by other strains of *Verrucomicrobiota* has been reported (17). The synthesized EPSs can adsorb macromolecular polysaccharides and other compounds that are present in the ocean, facilitating the formation of particles (20). These particles eventually sink and are transported to marine sediments, thereby contributing to carbon sequestration (20). The process by which bacteria synthesize glycogen and EPSs could affect the surrounding microbial community and the carbon cycle in a number of ways.

Finally, we investigated the differences in the strains’ abilities to synthesize and degrade polysaccharides under nitrogen-limited conditions. We found that while their ability to degrade polysaccharides decreased slightly under nitrogen-limited conditions, their polysaccharide synthesis capacity increased significantly under those conditions. In the absence of nitrogen (except when xylan or fucoidan was added), the strains tended to produce more EPSs, promoting biofilm formation. This experiment more closely simulates nitrogen-limited conditions in the ocean and helps us understand the differences between laboratory conditions and the natural environment in terms of *Thalassobacterium*’s carbon metabolism. This, in turn, provides a better understanding of how microorganisms contribute to the carbon cycle in natural environments. We also discovered that *Thalassobacterium* is able to grow in a nitrogen-free medium under aerobic conditions, a phenomenon previously reported but not observed in other *Verrucomicrobiota* or PVC superphylum strains (22). We hypothesize that these strains may increase biofilm formation under nitrogen-free conditions, creating a localized anaerobic environment that is conducive to nitrogen fixation. Additionally, the large amounts of glycogen stored by the strains may provide a continuous supply of energy for biofilm formation and nitrogen fixation. However, these hypotheses have not yet been experimentally verified. In the future, we may adopt research methods that have been used with other aerobic nitrogen-fixing bacteria in our study of *Verrucomicrobiota*, in the hope of providing a foundation for a more comprehensive understanding of their life processes and their contributions to the Earth’s biogeochemical cycles.

### Description of *Thalassobacterium* gen. nov., *Thalassobacterium maritimum* sp. nov. and *Thalassobacterium sedimentorum* sp. nov.

#### Description of *Thalassobacterium* gen. nov.

*Thalassobacterium* (Gr. fem. n. *thalassa*, the ocean; *bacterium* L. neut. n., a bacterium; *Thalassobacterium*, a microbe of the ocean).

The cells are Gram-stain-negative, spherical, mesophilic, and facultatively anaerobic. The major respiratory quinone is MK-7. The major polar lipids include diphosphatidylglycerol (DPG) and phosphatidylethanolamine (PE). The genus includes the species *Thalassobacterium maritimum* and *Thalassobacterium sedimentorum*.

#### Description of *Thalassobacterium maritimum* sp. nov.

*Thalassobacterium maritimum* (ma.ri′ti.mum. L. neut. adj. *maritimum*, of or belonging to the sea, marine).

A Gram-negative facultative anaerobic strain named SDUM461003^T^ was isolated from marine sediment samples. Strain SDUM461003^T^ was able to grow at 12–37°C (optimum, 25–35°C), 0.5–7% (wt/vol) NaCl (optimum, 2.0%), and pH 6.0–9.0 (optimum, 6.5–8.0). Cells of strain SDUM461003^T^ are spherical in shape, with a diameter of approximately 0.45–0.65 µm. White circular colonies were observed after 3 days of growth in R2A medium at 30°C. The Voges–Proskauer reaction and reactions for dextrin, d-cellobiose, sucrose, d-turanose, d-lactose, β-methyl-d-glucoside, d-salicin, α-d-glucose, d-mannose, d-fructose, d-galactose, l-fucose, l-rhamnose, d-serine, d-mannitol, glycerol, pectin, l-lactic acid, acetic acid, and sodium butyrate were positive, and the bacteria were positive for hydrolysis of agar, starch, and CM-cellulose and for oxidase and catalase enzymatic activity. Negative results were obtained for the hydrolysis of Tween 20, Tween 40, Tween 60, Tween 80, DNA, casein, and alginate. The major fatty acids of SDUM461003^T^ were C_16:0_, C_18:0_, iso-C_14:0_, anteiso-C_15:0_, and C_18:1_ ω9c. The major polar lipids of SDUM461003^T^ were phosphatidylglycerol, diphosphatidylglycerol, phosphatidylethanolamine, and aminophospholipids.

#### Description of *Thalassobacterium sedimentorum* sp. nov.

*Thalassobacterium sedimentorum* (se.di.men.to′rum. L. gen. neut. pl. n. *sedimentorum*, of sediments).

A Gram-negative facultative anaerobic strain named SDUM461004^T^ was isolated. It was able to grow at 20–40°C (optimum, 30–33°C), 0.5–5% (wt/vol) NaCl (optimum, 2.0–2.5%), and pH 6.0–9.0 (optimum, 6.5–7.0). Cells of strain SDUM461004^T^ are spherical in shape, with a diameter of approximately 0.40–0.75 µm. White circular colonies were observed after 3 days of growth in R2A medium at 30°C. The Voges–Proskauer reaction and reactions for d-maltose, d-cellobiose, d-lactose, α-d-glucose, d-mannose, d-fructose, d-galactose, l-fucose, l-rhamnose, d-mannitol, glycerol, d-glucuronic acid, acetic acid, and sodium butyrate were positive. The bacteria were negative for the hydrolysis of agar, starch, CM-cellulose, Tween 20, Tween 40, Tween 60, Tween 80, DNA, casein, and alginate, as well as for catalase enzymatic activity. The major fatty acids of SDUM461003^T^ were C_14:0_, C_16:0_, C_18:0_, iso-C_14:0_, anteiso-C_15:0_, and C_18:1_ ω9c. The major polar lipids of SDUM461003^T^ were diphosphatidylglycerol and phosphatidylethanolamine.

## MATERIALS AND METHODS

### Isolation and identification of bacteria

SDUM461003^T^ and SDUM461004^T^ were isolated from marine sediments in Weihai, China (36°58′37″ N, 122°2′37″ E). After the sediment was collected, 1 g of the sample was added to 100 mL of sterilized seawater containing glass beads for low-temperature shock. After gradient dilution, 200 µL of the diluent was applied to 1/10 marine agar 2216 (MA). After 2 weeks of culture, a single colony was selected and placed in marine broth 2216 medium for pure culture, and the 16S rRNA gene was extracted for identification.

The nearly complete 16S rRNA gene sequences of strains SDUM461003^T^ and SDUM461004^T^ were obtained by cloning into a pMD18-T vector (Takara). The primers used were M13F and M13R, and the PCR conditions were predenaturation at 94°C for 6 min and 31 cycles of denaturation at 94°C for 45 s, annealing at 54°C for 45 s, and extension at 72°C for 100 s. The obtained 16S rRNA gene sequences of the strains were compared with those found in the NCBI (https://www.ncbi.nlm.nih.gov/) and EzBioCloud (https://www.ezbiocloud.net/) databases to determine the strains’ taxonomic status. Phylogenetic trees were constructed via the neighbor-joining (NJ), maximum likelihood (ML; model GTR + G + I), and maximum parsimony (MP) algorithms in MEGA version 11.0 (24–26). The bootstrap value was set to 1000.

The related type strains *Coraliomargarita sinensis* WN38^T^ (published by our laboratory) (27) and *Coraliomargarita akajimensis* KCTC 12865^T^ (purchased from the Korean Collection for Type Cultures, KCTC) were used as references.

### Genomic analysis

DNA was extracted from strains SDUM461003^T^ and SDUM461004^T^ using a DNA kit (Takara) and sequenced by Novogene Bioinformatics Technology Co., Ltd. (Beijing, China) on the Illumina NovaSeq 6000 platform. The obtained sequences were filtered and assembled using fastp software and SOAPdenovo software v2.04 (28). The average nucleotide identity (ANI) value was calculated via the online tool EzBioCloud (https://www.ezbiocloud.net/tools/ani) (29). The metabolic pathways of the genomes were annotated in the KEGG database (https://www.genome.jp/kegg/). The antibiotic resistance of the strains was analyzed using the CARD database (https://card.mcmaster.ca/home) (30). The carbohydrate-active enzymes of the genomes were annotated in dbCAN (https://bcb.unl.edu/dbCAN2/blast.php) (31). The core–pangenome was analyzed via the bacterial pangenome analysis pipeline (IPGA-Version-1.09) (32). Potential polysaccharide degradation gene clusters were annotated using the method of Lu et al. (33).

### Phenotypic, physiological, and biochemical characteristics of the strains

Gram-stained samples of strains SDUM461003^T^ and SDUM461004^T^ were prepared and examined using a Gram-staining kit (Hopebio, Qingdao). The catalase activity was determined by observing the production of bubbles in 3% (vol/vol) H_2_O_2_ solution. An oxidase experiment was conducted using an oxidase reagent (bioMérieux). The growth of the strains at various temperatures (0, 4, 10, 12, 15, 20, 25, 28, 30, 33, 35, 37, 40, and 43°C) was tested on R_2_A plates prepared from stale seawater. The salinity range of strain growth was tested at NaCl concentrations of 0, 0.5, 1.0, 1.5, 2.0, 2.5, 3.0, 4.0, 5.0, 7.0, 8.0, and 10.0% (wt/vol) in medium containing 0.23% MgCl_2_, 0.32% MgSO_4_, 0.12% CaCl_2_, 0.07% KCl, 0.02% NaHCO_3_, 0.5% peptone, 0.1% yeast extract, and 2% (wt/vol) agar. The growth of the strains was measured at pH values of 5.5, 6.0, 6.5, 7.0, 7.5, 8.0, 8.5, 9.0, and 9.5 in marine broth 2216 (MB; BD). The pH of the medium at 30°C was adjusted using buffers containing MES (pH 5.5–6.0), PIPES (pH 6.5–7.0), HEPES (pH 7.5–8.0), Tricine (pH 8.5), and CAPSO (pH 9.0–14.0). The morphology and diameter of the cells were observed by light microscopy (E600, Nikon), scanning electron microscopy (Nova Nano-SEM450; FEI), and transmission electron microscopy (JEM-1200, JEOL). The ability to hydrolyze DNA, CM-cellulose, starch, alginate, casein, and Tween 20, 40, 60, and 80 was determined according to the methods of Smibert (34). The strains were grown in mineral medium (0.3 g sodium pyruvate, 1 g yeast extract, 20 g agar, and 1,000 mL stale seawater) for 5 days at 30°C, and the plates were then flooded with Lugol’s stain solution. A clear zone forming around the agar indicated agar-degrading activity (35). The antibiotic susceptibility of the strains was tested on R_2_A using the disc diffusion method described by Bauer (36), and their susceptibility to individual antibiotics was determined according to the guidelines provided by the Clinical and Laboratory Standards Institute (CLSI). The utilization of starch, chondroitin sulfate, xylan, and fucoidan by the strains was measured in liquid medium. The control was a simplified liquid medium (1 g/L urea, 1 g/L glucose, and 1 ml/L Wolfe’s mineral mixture dissolved in artificial seawater). The nitrogen-deficient medium did not contain urea. A significant increase in the OD_600_ of the cultures grown in medium supplemented with various polysaccharides (2 g/L), compared with the OD_600_ of the control culture, indicated that the strain was able to degrade the corresponding polysaccharides and use them for growth. In these experiments, the inoculum size was 5%, the cultures were grown in artificial seawater overnight, and the OD_600_ was adjusted to approximately 0.4 before addition of the polysaccharides. The biofilm content was detected by crystal violet staining (37). The motility of the strains was determined on modified MA plates (0.3% agar). The physiological and biochemical characteristics of the strains were tested using API 20E kits (bioMérieux) and Biolog GEN III MicroPlates according to the manufacturer’s instructions.

### Chemotaxonomic analyses

SDUM461003^T^ and SDUM461004^T^ and the reference strains were cultured in MB for 5 days at 30°C. For polar lipid composition analysis, the components were extracted and spilled off in two different directions on a TLC board according to the method of Minnikin (35). The respiratory quinones of strains SDUM461003^T^ and SDUM461004^T^ were determined using high-performance liquid chromatography (HPLC) (38). Fatty acids were extracted and detected by using high-performance gas chromatography (39).

## Data Availability

The GenBank accession number for the 16S rRNA gene sequence of strain SDUM461003^T^ (=MCCC 1H01364^T^ =KCTC 92748^T^) is OR467175, and the draft genome has been deposited in GenBank under the accession number JARXHW000000000. The GenBank accession number for the 16S drRNA gene sequence of strain SDUM461004^T^ (=MCCC 1H01402^T^=KCTC 92749^T^) is OR467180, and the draft genome has been deposited in GenBank under the accession number JARXIC000000000.

## SUPPLEMENTAL MATERIAL

Fig S1 mbio.00305-25-s0001.tif Scanning electron microscopy and transmission electron microscopy images.

Fig S2 mbio.00305-25-s0002.tif Polar lipid profile of strains visualized using biphasic TLC with phosphomolybdic acid staining.

Fig S3 mbio.00305-25-s0003.tiff Composition and location of PUL-Likes and CGCs on the genome of different strains.

Supplemental Figure legends mbio.00305-25-s0004.docx Legends for Fig. S1-S3.

Supplemental Tables mbio.00305-25-s0005.docx Tables S1-S5

ASM does not own the copyrights to Supplemental Material that may be linked to, or accessed through, an article. The authors have granted ASM a non-exclusive, world-wide license to publish the Supplemental Material files. Please contact the corresponding author directly for reuse.

## References

[1] Schmidt TM. 2019. Encyclopedia of microbiology. Academic Press.

[2] Rappé MS, Giovannoni SJ. 2003. The uncultured microbial majority. Annu Rev Microbiol 57:369–394. doi:10.1146/annurev.micro.57.030502.090759 14527284

[3] Joseph SJ, Hugenholtz P, Sangwan P, Osborne CA, Janssen PH. 2003. Laboratory cultivation of widespread and previously uncultured soil bacteria. Appl Environ Microbiol 69:7210–7215. doi:10.1128/AEM.69.12.7210-7215.2003 14660368 PMC309996

[4] Kanokratana P, Chanapan S, Pootanakit K, Eurwilaichitr L. 2004. Diversity and abundance of bacteria and archaea in the Bor Khlueng hot spring in Thailand. J Basic Microbiol An Int J Biochem Physiol Genet Morphol Ecol Microorg 44:430–444. doi:10.1002/jobm.200410388 15558824

[5] Freitas S, Hatosy S, Fuhrman JA, Huse SM, Welch DBM, Sogin ML, Martiny AC. 2012. Global distribution and diversity of marine Verrucomicrobia. ISME J 6:1499–1505. doi:10.1038/ismej.2012.3 22318305 PMC3400412

[6] Sichert A, Corzett CH, Schechter MS, Unfried F, Markert S, Becher D, Fernandez-Guerra A, Liebeke M, Schweder T, Polz MF, Hehemann J-H. 2020. Verrucomicrobia use hundreds of enzymes to digest the algal polysaccharide fucoidan. Nat Microbiol 5:1026–1039. doi:10.1038/s41564-020-0720-2 32451471

[7] Orellana LH, Francis TB, Ferraro M, Hehemann J-H, Fuchs BM, Amann RI. 2022. Verrucomicrobiota are specialist consumers of sulfated methyl pentoses during diatom blooms. ISME J 16:630–641. doi:10.1038/s41396-021-01105-7 34493810 PMC8857213

[8] Naumoff DG, Dedysh SN. 2018. Bacteria from poorly studied phyla as a potential source of new enzymes: β-galactosidases from planctomycetes and verrucomicrobia. Microbiology (Reading, Engl) 87:796–805. doi:10.1134/S0026261718060127

[9] Shieh WY, Jean WD. 1998. Alterococcus agarolyticus, gen.nov., sp.nov., a halophilic thermophilic bacterium capable of agar degradation. Can J Microbiol 44:637–645. doi:10.1139/cjm-44-7-637 9783423

[10] Hansell DA. 2013. Recalcitrant dissolved organic carbon fractions. Ann Rev Mar Sci 5:421–445. doi:10.1146/annurev-marine-120710-100757 22881353

[11] Kim TK, Hewavitharana AK, Shaw PN, Fuerst JA. 2006. Discovery of a new source of rifamycin antibiotics in marine sponge actinobacteria by phylogenetic prediction. Appl Environ Microbiol 72:2118–2125. doi:10.1128/AEM.72.3.2118-2125.2006 16517661 PMC1393243

[12] Van Anh C, Kang JS, Yang J-W, Kwon J-H, Heo C-S, Lee H-S, Shin HJ. 2023. Rifamycin-related polyketides from a marine-derived bacterium Salinispora arenicola and their cytotoxic activity. Mar Drugs 21:494. doi:10.3390/md21090494 37755107 PMC10532523

[13] Lu DC, Wang FQ, Amann RI, Teeling H, Du ZJ. 2023. Epiphytic common core bacteria in the microbiomes of co-located green (Ulva), brown (Saccharina) and red (Grateloupia, Gelidium) macroalgae. Microbiome 11:126. doi:10.1186/s40168-023-01559-1 37264413 PMC10233909

[14] Synytsya A, Čopíková J, Kim WJ. 2015. Cell wall polysaccharides of marine algae BT, p 543–590. In Kim SK (ed), Springer handbook of marine biotechnology. Springer, Berlin, Germany.

[15] Bünger W, Jiang X, Müller J, Hurek T, Reinhold-Hurek B. 2020. Novel cultivated endophytic Verrucomicrobia reveal deep-rooting traits of bacteria to associate with plants. Sci Rep 10:8692. doi:10.1038/s41598-020-65277-6 32457320 PMC7251102

[16] Zhang Y-S, Zhang Y-Q, Zhao X-M, Liu X-L, Qin Q-L, Liu N-H, Xu F, Chen X-L, Zhang Y-Z, Li P-Y. 2024. Metagenomic insights into the dynamic degradation of brown algal polysaccharides by kelp-associated microbiota. Appl Environ Microbiol 90:e02025-23. doi:10.1128/aem.02025-23 38259074 PMC10880675

[17] Khadem AF, van Teeseling MCF, van Niftrik L, Jetten MSM, Op den Camp HJM, Pol A. 2012. Genomic and physiological analysis of carbon storage in the verrucomicrobial methanotroph “Ca. Methylacidiphilum fumariolicum” SolV. Front Microbiol 3:345. doi:10.3389/fmicb.2012.00345 23060867 PMC3460235

[18] Sartorio MG, Pardue EJ, Scott NE, Feldman MF. 2023. Human gut bacteria tailor extracellular vesicle cargo for the breakdown of diet- and host-derived glycans. Proc Natl Acad Sci U S A 120:e2306314120. doi:10.1073/pnas.2306314120 37364113 PMC10319031

[19] Flemming HC, Wingender J. 2010. The biofilm matrix. Nat Rev Microbiol 8:623–633. doi:10.1038/nrmicro2415 20676145

[20] Engel A, Thoms S, Riebesell U, Rochelle-Newall E, Zondervan I. 2004. Polysaccharide aggregation as a potential sink of marine dissolved organic carbon. Nature 428:929–932. doi:10.1038/nature02453 15118723

[21] Hartmann A, Fu H, Burris RH. 1986. Regulation of nitrogenase activity by ammonium chloride in Azospirillum spp. J Bacteriol 165:864–870. doi:10.1128/jb.165.3.864-870.1986 3081492 PMC214508

[22] Wang D, Xu A, Elmerich C, Ma LZ. 2017. Biofilm formation enables free-living nitrogen-fixing rhizobacteria to fix nitrogen under aerobic conditions. ISME J 11:1602–1613. doi:10.1038/ismej.2017.30 28338674 PMC5520150

[23] Inomura K, Bragg J, Follows MJ. 2017. A quantitative analysis of the direct and indirect costs of nitrogen fixation: a model based on Azotobacter vinelandii*.* ISME J 11:166–175. doi:10.1038/ismej.2016.97 27740611 PMC5315487

[24] Tamura K, Stecher G, Kumar S. 2021. MEGA11: Molecular Evolutionary Genetics Analysis version 11. Mol Biol Evol 38:3022–3027. doi:10.1093/molbev/msab120 33892491 PMC8233496

[25] Fitch WM. 1971. Toward defining the course of evolution: minimum change for a specific tree topology. Syst Biol 20:406–416. doi:10.1093/sysbio/20.4.406

[26] Saitou N, Nei M. 1987. The neighbor-joining method: a new method for reconstructing phylogenetic trees. Mol Biol Evol 4:406–425. doi:10.1093/oxfordjournals.molbev.a040454 3447015

[27] Zhou LY, Wang NN, Mu DS, Liu Y, Du ZJ. 2019. Coraliomargarita sinensis sp. nov., isolated from a marine solar saltern. Int J Syst Evol Microbiol 69:701–707. doi:10.1099/ijsem.0.003205 30694173

[28] Li R, Yu C, Li Y, Lam T-W, Yiu S-M, Kristiansen K, Wang J. 2009. SOAP2: an improved ultrafast tool for short read alignment. Bioinformatics 25:1966–1967. doi:10.1093/bioinformatics/btp336 19497933

[29] Yoon SH, Ha SM, Lim J, Kwon S, Chun J. 2017. A large-scale evaluation of algorithms to calculate average nucleotide identity. Antonie Van Leeuwenhoek 110:1281–1286. doi:10.1007/s10482-017-0844-4 28204908

[30] Alcock BP, Huynh W, Chalil R, Smith KW, Raphenya AR, Wlodarski MA, Edalatmand A, Petkau A, Syed SA, Tsang KK, et al.. 2023. CARD 2023: expanded curation, support for machine learning, and resistome prediction at the comprehensive antibiotic resistance database. Nucleic Acids Res 51:D690–D699. doi:10.1093/nar/gkac920 36263822 PMC9825576

[31] Zheng J, Ge Q, Yan Y, Zhang X, Huang L, Yin Y. 2023. dbCAN3: automated carbohydrate-active enzyme and substrate annotation. Nucleic Acids Res 51:W115–W121. doi:10.1093/nar/gkad328 37125649 PMC10320055

[32] Chaudhari NM, Gupta VK, Dutta C. 2016. BPGA- an ultra-fast pan-genome analysis pipeline. Sci Rep 6:24373. doi:10.1038/srep24373 27071527 PMC4829868

[33] Gerhardt P. 1994. Methods for general and molecular bacteriology. Available from: https://api.semanticscholar.org/CorpusID:83257884

[34] Alvarado R, Leiva S. 2017. Agar-degrading bacteria isolated from Antarctic macroalgae. Folia Microbiol (Praha) 62:409–416. doi:10.1007/s12223-017-0511-1 28283945

[35] Minnikin DE, O’Donnell AG, Goodfellow M, Alderson G, Athalye M, Schaal A, Parlett JH. 1984. An integrated procedure for the extraction of bacterial isoprenoid quinones and polar lipids. J Microbiol Methods 2:233–241. doi:10.1016/0167-7012(84)90018-6

[36] Bauer AW, Kirby WM, Sherris JC, Turck M. 1966. Antibiotic susceptibility testing by a standardized single disk method. Am J Clin Pathol 45:493–496. 5325707

[37] Ma L, Jackson KD, Landry RM, Parsek MR, Wozniak DJ. 2006. Analysis of Pseudomonas aeruginosa conditional psl variants reveals roles for the psl polysaccharide in adhesion and maintaining biofilm structure postattachment. J Bacteriol 188:8213–8221. doi:10.1128/JB.01202-06 16980452 PMC1698210

[38] Kroppenstedt RM. 1982. Separation of bacterial menaquinones by HPLC using reverse phase (RP18) and a silver loaded ion exchanger as stationary phases. J Liq Chromatogr 5:2359–2367. doi:10.1080/01483918208067640

[39] Athalye M, Noble WC, Minnikin DE. 1985. Analysis of cellular fatty acids by gas chromatography as a tool in the identification of medically important coryneform bacteria. J Appl Bacteriol 58:507–512. doi:10.1111/j.1365-2672.1985.tb01491.x 3924876

