# Correction for Tan et al., “Insights into the physiological and metabolic features of *Thalassobacterium*, a novel genus of *Verrucomicrobiota* with the potential to drive the carbon cycle”

**DOI:** 10.1128/mbio.00907-26

**Published:** 2026-05-18

**Authors:** Xin-Yun Tan, Xin-Jiang Liu, De-Chen Lu, Yu-Qi Ye, Xin-Yu Liu, Fan Yu, Hui Yang, Fan Li, Zong-Jun Du, Meng-Qi Ye

## AUTHOR CORRECTION

Volume 16, no. 4, e00305-25, 2025, https://doi.org/10.1128/mbio.00305-25. To comply with the taxonomic validation requirements of *International Journal of Systematic and Evolutionary Microbiology* (IJSEM) and Rule 27 of the International Code of Nomenclature of Prokaryotes (ICNP), we are providing the type species designation to the published record of *Thalassobacterium* gen. nov. No changes to the physiological characterization, genomic analysis, experimental results, or scientific conclusions of the original article are made herein.

Discussion section, “Description of *Thalassobacterium* gen. nov.”: The following should be added as the last sentence. “The type species is *Thalassobacterium maritimum*.”

Discussion section, “Description of *Thalassobacterium maritimum* sp. nov.”: The following should be added as the last sentence. “The type strain is SDUM461003ᵀ (=MCCC 1H01364ᵀ =KCTC 92748ᵀ).”

Discussion section, “Description of *Thalassobacterium sedimentorum* sp. nov.” : The following should be added as the last sentence. “The type strain is SDUM461004ᵀ (=MCCC 1H01402ᵀ =KCTC 92749ᵀ).”

