# Supplementary figures and images for: Insights into the physiological and metabolic features of Thalassobacterium, a novel genus of Verrucomicrobiota with the potential to drive the carbon cycle
Source: mBio. 2025 Mar 20;16(4):e00305-25. doi: 10.1128/mbio.00305-25 (PMC11980603; doi:10.1128/mbio.00305-25)

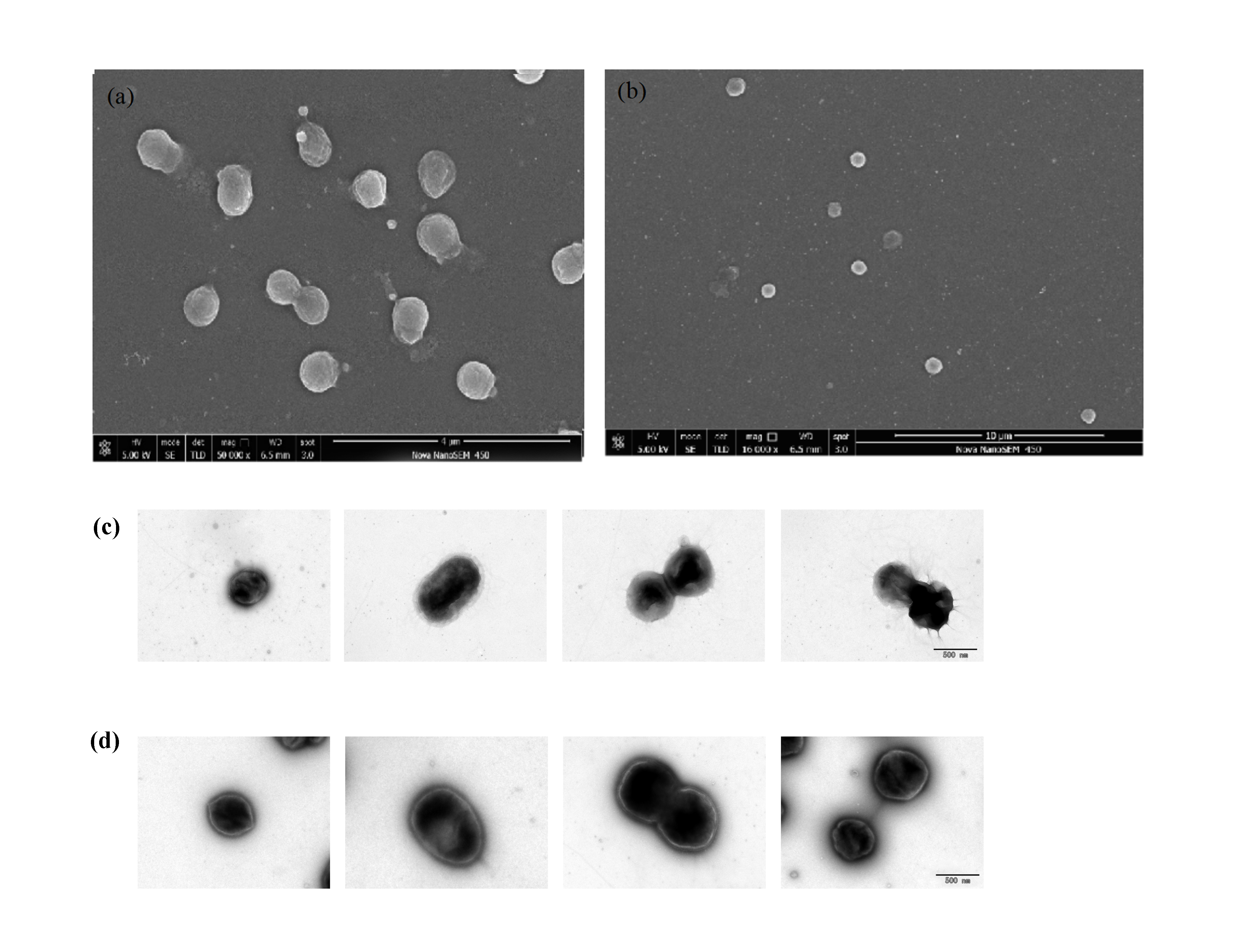

Supplement: Fig S1 — Scanning electron microscopy and transmission electron microscopy images. [file mbio.00305-25-s0001.tif]

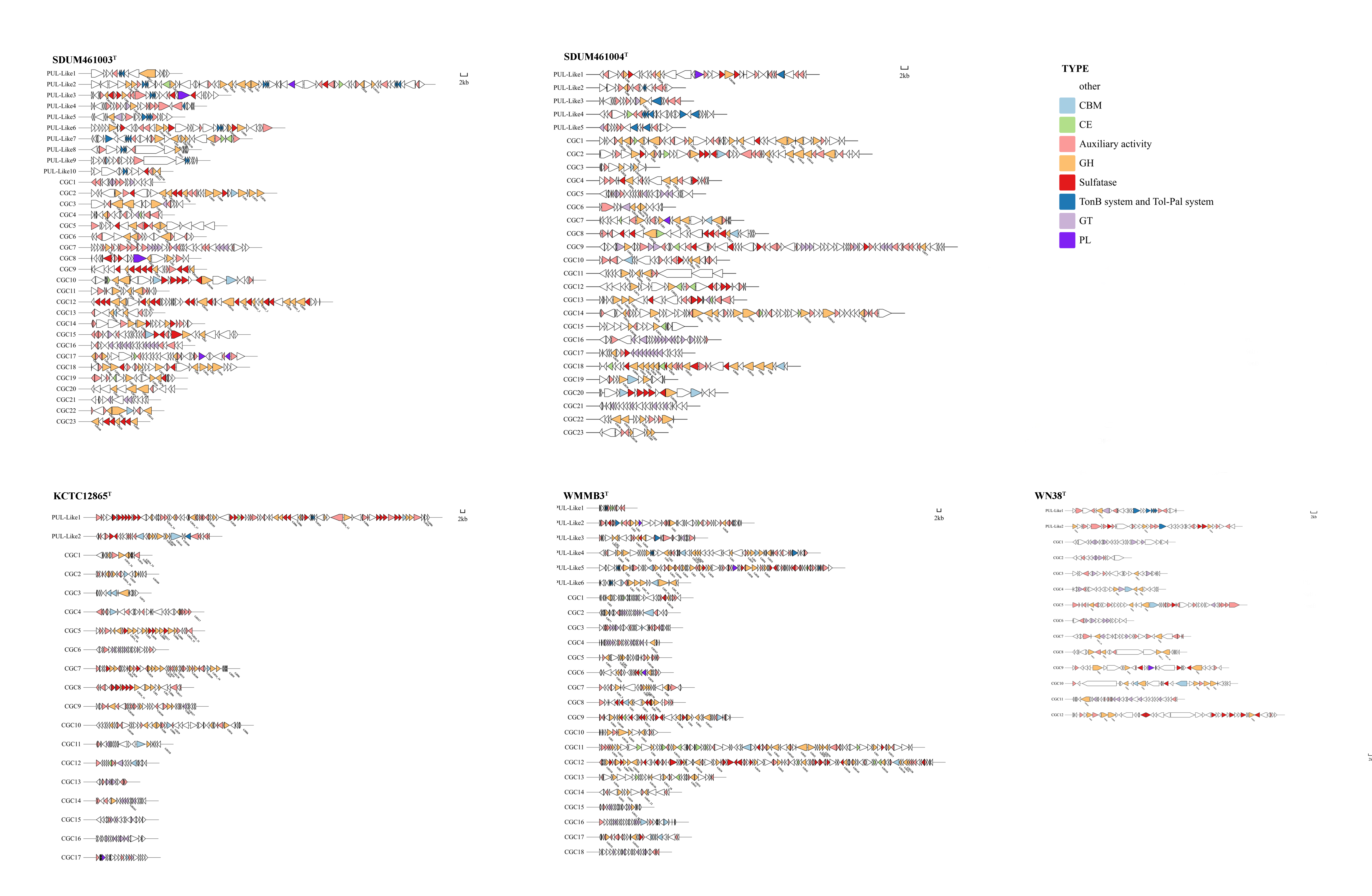

Supplement: Fig S3 — Composition and location of PUL-Likes and CGCs on the genome of different strains. [file mbio.00305-25-s0003.tiff]
