# Supplementary material for: Insights into the physiological and metabolic features of Thalassobacterium, a novel genus of Verrucomicrobiota with the potential to drive the carbon cycle
Source: mBio. 2025 Mar 20;16(4):e00305-25. doi: 10.1128/mbio.00305-25 (PMC11980603; doi:10.1128/mbio.00305-25)
Supplement: Supplemental Figure legends — Legends for Fig. S1-S3. [file mbio.00305-25-s0004.docx]

**Figure S1:** (a) Cell morphology of strain SDUM461003^T^ shown by scanning electron microscopy. Scale bar, 4 µm. (b) Cell morphology of strain SDUM461004^T^ shown by scanning electron microscopy. Scale bar, 10 µm. (c) Cell morphology of strain SDUM461003^T^ shown by transmission electron microscopy. Scale bar, 500 nm. (d) Cell morphology of strain SDUM461004^T^ shown by transmission electron microscopy. Scale bar, 600 nm.

**Figure S2**: The figure shows the polar lipid profile of strains visualized using biphasic thin-layer chromatography (TLC) with phosphomolybdic acid staining. a, SDUM461003^T^; b, SDUM461004^T^; c, C. sinensis WN38^T^; d, C. akajimensis KCTC 12865^T^. PG: phosphatidylglycerol, PE: phosphatidylethanolamine, DPG: Diphosphatidylglycerol, PL: unidentified phospholipid, APL: aminophospholipid, L: unidentified lipids.

**Figure S3**. Composition and location of PUL-Likes and CGCs on the genome of different strains. The bands from bottom to top of each strain indicated the type of polysaccharides degradation gene clusters, the composition of gene clusters, and the distribution location of its protein components.
