# Supplementary material for: Insights into the physiological and metabolic features of Thalassobacterium, a novel genus of Verrucomicrobiota with the potential to drive the carbon cycle
Source: mBio. 2025 Mar 20;16(4):e00305-25. doi: 10.1128/mbio.00305-25 (PMC11980603; doi:10.1128/mbio.00305-25)
Supplement: Supplemental Tables — Tables S1-S5 [file mbio.00305-25-s0005.docx]

**Table S1**. Comparisons of the average nucleotide identity (ANI) and average amino acid identity (AAI) values between strains SDUM461003^T^, SDUM461004^T^, and related type strains.

| Strain 1 | Strain 2 | ANI (%) | AAI (%) |
| --- | --- | --- | --- |
| SDUM461003^T^ | SDUM461004^T^ | 82.6 | 85.3 |
| SDUM461003^T^ | *C. akajimensis* KCTC 12865^T^ | 73.1 | 69.5 |
| SDUM461003^T^ | *C. sinensis* WN38^T^ | 73.5 | 71.6 |
| SDUM461003^T^ | *C. parva* WMMB3^T^ | 71.6 | 70.5 |
| SDUM461004^T^ | *C. akajimensis* KCTC 12865^T^ | 72.8 | 69.6 |
| SDUM461004^T^ | *C. sinensis* WN38^T^ | 73.1 | 71.5 |
| SDUM461004^T^ | *C. parva* WMMB3^T^ | 71.2 | 70.4 |

**Table S2**.Genome data of strains SDUM461003^T^, SDUM461004^T^, and other type strains.

| Features | 1 | 2 | 3 | 4 | 5 |
| --- | --- | --- | --- | --- | --- |
| Genome size (bp) | 4,917,260 | 4,598,784 | 3,750,771 | 3,569,655 | 4,497,538 |
| CheckM completeness (%) |  |  | 99.3 | 93.5 | 96.7 |
| CheckM contamination (%) |  |  | 0.1 | 2.1 | 4.1 |
| Genome coverage | 323X | 263X | ND | 100X | 160X |
| Contig count | 317 | 164 | 1 | 36 | 27 |
| N50 (bp) | 77,583 | 128,435 | 3,750,771 | 367,257 | 357,974 |
| Genes | 4,092 | 3,778 | 3,076 | 3,058 | 3,714 |
| tRNAs | 46 | 40 | 46 | 41 | 45 |
| rRNAs | 3 | 3 | 6 | 3 | 3 |
| ncRNAs | 4 | 4 | 4 | 4 | 4 |
| DNA G+C content (%) | 52.2 | 50.3 | 53.9 | 55.6 | 56.0 |
| GenBank assembly accession | JARXHW000000000 | JARXIC000000000 | NC_014008 | NZ_QHJQ01000000 | NZ_JAPZEI000000000 |

Strains: 1, SDUM461003^T^; 2, SDUM461004^T^; 3, *C. akajimensis* KCTC 12865^T^; 4, *C. sinensis* WN38^T^; 5, *C. parva* WMMB3^T^.

**Table S3**.The ability of SDUM461003^T^ and SDUM461004^T^ to utilize carbon sources was tested by BIOLOG

|  | D-Galacturonic Acid | L-Galactonic Acid Lactone | Dextrin | D-Cellobiose | Sucrose | D-Turanose | Rifamycin SV | D-Lactose | β-Methyl-D-Glucoside | D-Salicin | Lincomycin |
| --- | --- | --- | --- | --- | --- | --- | --- | --- | --- | --- | --- |
| SDUM461003 | w | w | + | + | + | + | w | + | + | + | - |
| SDUM461004 | W | W | - | + | - | - | + | + | + | + | + |
|  | N-Acetyl-D-Glucosamine | N-Acetyl-D- Galactosamine | α-D-Glucose | D-Mannose | D-Fructose | D-Galactose | L-Fucose | L-Rhamnose | 1% Sodium Lactate | D-Serine | D-Glucuronic Acid |
| SDUM461003 | + | + | + | + | + | + | + | + | + | + | - |
| SDUM461004 | + | + | + | + | + | + | + | + | + | W | + |
|  | Sodium Butyrate | D-Lactic Acid Methyl Ester | Pectin | Glucuronamide | Vancomycin | Methyl Pyruvate | Glycerol | L-Lactic Acid | D-Malic Acid | Nalidixic Acid | Minocycline |
| SDUM461003 | + | + | + | + | + | + | + | + | + | + | - |
| SDUM461004 | + | - | - | + | + | + | + | + | - | + | W |
|  | Lithium Chloride | Acetoacetic Acid | Acetic Acid | Aztreonam | D-Mannitol | D-Fucose | D-Maltose | Fusidic Acid |  |  |  |
| SDUM461003 | + | + | + | + | + | + | - | - |  |  |  |
| SDUM461004 | + | W | + | + | + | + | + | + |  |  |  |

**Table S4.** The antibiotic sensitivity test of strain SDUM461003^T^ and strain SDUM461004^T^, +, indicates that the strain is resistant to antibiotics; -, indicates that the strain is sensitive to antibiotics.

|  | concentration | SDUM461003 | SDUM461004 |
| --- | --- | --- | --- |
| ampicillin | 10 | - | - |
| polymyxin B | 300 | - | + |
| erythromycin | 15 | + | + |
| kanamycin | 30 | + | + |
| clarithromycin | 15 | + | + |
| Rifampin | 5 | + | + |
| streptomycin | 10 | + | + |
| lincomycin | 2 | + | + |
| chloramphenicol | 30 | + | + |
| norfloxacin | 30 | + | - |
| penicillin | 10 | - | + |
| gentamycin | 10 | - | + |
| tetracycline | 30 | + | + |
| carbenicillin | 100 | - | + |
| ceftriaxone | 30 | - | + |
| Cefotaxime Sodium | 30 | - | + |
| tobramycin | 10 | + | + |
| vancomycin | 30 | + | + |
| neomycin | 30 | + | + |
| ofloxacin | 5 | + | - |

**Table S5**. Cellular fatty acid composition (%) of strains SDUM461003^T^, SDUM461004^T^ and related type strains.

| Fatty acid | 1 | 2 | 3 | 4 |
| --- | --- | --- | --- | --- |
| Straight-chain fatty acids |  |  |  |  |
| C_12:0_ | TR | 1.0 | TR | TR |
| C_14:0_ | **14.7** | **11.1** | **18.8** | **11.4** |
| C_16:0_ | **7.5** | **10.5** | 4.4 | 4.6 |
| C_17:0_ | 1.4 | 2.9 | TR | TR |
| C_18:0_ | **15.1** | **11.0** | **24.7** | **19.9** |
| C_19:0_ | TR | TR | 1.1 | TR |
| C_20:0_ | 1.1 | TR | **8.1** | 4.8 |
| Branched chain |  |  |  |  |
| iso-C_14:0_ | **15.2** | **14.9** | **14.0** | **18.4** |
| anteiso-C_15:0_ | **6.7** | **6.3** | 2.3 | 2.8 |
| iso-C_16:0_ | 1.9 | 1.8 | 2.0 | 1.4 |
| iso-C_18:0_ | 1.4 | － | TR | 1.1 |
| Monounsaturated |  |  |  |  |
| C_17:1_ ω8c | 1.5 | 2.0 | TR | TR |
| C_18:1_ ω9c | **27.1** | **25.5** | **14.7** | **23.7** |
| C_18:3_ ω6c | － | 4.2 | － | TR |
| Hydroxy fatty acids |  |  |  |  |
| C_12:0_ 3-OH | TR | TR | 2.8 | 2.7 |
| iso-C_14:0_ 3-OH | 1.1 | 2.2 | － | 2.4 |
| C_16:0_ 3-OH | － | TR | 1.0 | － |
| Summed feature* |  |  |  |  |
| 3 | **29.17** | **27.93** | **19.05** | **21.13** |
| 8 | － | － | TR | 2.17 |

Strains: 1, SDUM461003^T^; 2, SDUM461004^T^; 3, *C. akajimensis* KCTC 12865^T^; 4, *C. sinensis* WN38^T^. All data were obtained in this study. Fatty acids (>5%) were marked in bold. The figure in the table represents the percentages. *Summed features represent groups of two or three fatty acids that could not be separated by GLC with the MIDI system. Summed Features 8 consisted of C_18:1_ω7c/C_18:1_ω6c. Symbols: －, Not detected; TR, trace (< 1%).
